# Transcription factor *StWRKY1* regulates phenylpropanoid metabolites conferring late blight resistance in potato

**DOI:** 10.1093/jxb/erv434

**Published:** 2015-09-28

**Authors:** Kalenahalli N. Yogendra, Arun Kumar, Kobir Sarkar, Yunliang Li, Doddaraju Pushpa, Kareem A. Mosa, Raj Duggavathi, Ajjamada C. Kushalappa

**Affiliations:** ^1^Department of Plant Science, McGill University, Ste Anne de Bellevue, Quebec, Canada; ^2^Department of Animal Science, McGill University, Ste Anne de Bellevue, Quebec, Canada

**Keywords:** Hydroxycinnamic acid amides, *Phytophthora infestans*, quantitative resistance, secondary cell wall thickening, transcription factors

## Abstract

Quantitative resistance is polygenically controlled and durable, but the underlying molecular and biochemical mechanisms are poorly understood. Secondary cell wall thickening is a critical process in quantitative resistance, regulated by transcriptional networks. This paper provides compelling evidence on the functionality of StWRKY1 transcription factor, in a compatible interaction of potato–*Phytophthora infestans*, to extend our knowledge on the regulation of the metabolic pathway genes leading to strengthening the secondary cell wall. A metabolomics approach was used to identify resistance-related metabolites belonging to the phenylpropanoid pathway and their biosynthetic genes regulated by *StWRKY1*. The *StWRKY1* gene in resistant potato was silenced to decipher its role in the regulation of phenylpropanoid pathway genes to strengthen the secondary cell wall. Sequencing of the promoter region of *StWRKY1* in susceptible genotypes revealed the absence of heat shock elements (HSEs). Simultaneous induction of both the heat shock protein (sHSP17.8) and StWRKY1 following pathogen invasion enables functioning of the latter to interact with the HSE present in the resistant *StWRKY1* promoter region. EMSA and luciferase transient expression assays further revealed direct binding of StWRKY1 to promoters of hydroxycinnamic acid amide (HCAA) biosynthetic genes encoding 4-coumarate:CoA ligase and tyramine hydroxycinnamoyl transferase. Silencing of the *StWRKY1* gene was associated with signs of reduced late blight resistance by significantly increasing the pathogen biomass and decreasing the abundance of HCAAs. This study provides convincing evidence on the role of *StWRKY1* in the regulation of downstream genes to biosynthesize HCAAs, which are deposited to reinforce secondary cell walls.

## Highlight

StWRKY1 transcription factor mediates secondary cell wall thickening in potato by regulating expression of tyramine-related hydroxycinnamic acid amide biosynthetic genes, thus contributing to resistance to late blight disease.

## Introduction

Late blight caused by *Phytophthora infestans* (Mont.) de Bary is the most destructive disease of potato. It reduces plant biomass or even may kill the plant, thus decreasing crop quantity and quality (Fry, 2008). The resistance in potato can be improved by selecting resistance genes that are induced following pathogen invasion, either by molecular breeding or *cis*/*trans* genetic engineering. Plant responses to stress are complex and involve numerous physiological, molecular, and cellular adaptations (Rejeb *et al.*, 2014). The plant cell wall is one of the barriers that pathogens need to overcome to successfully colonize plant tissues. Cell wall thickening reduces the spread of pathogens in plants beyond the site of infection, reducing lesion expansion (Dangl *et al.*, 2013). The biosynthesis of the secondary cell wall is a developmental process that involves the co-ordinated expression of secondary cell wall biosynthetic genes regulated by a cascade of transcription factors (Cosgrove, 2005). Understanding such processes is vital for the manipulation of secondary cell wall thickening to resist pathogen development in plants (War *et al.*, 2012).

Plants have evolved intricate strategies to recognize pathogen infection and devise effective defence responses (Jones and Dangl, 2006). The first line of defence following infection involves the recognition of pathogen-associated molecular patterns (PAMPs), which trigger basal levels of plant defence responses referred to as PAMP-triggered immunity (PTI). To combat PTI, pathogens produce haustoria inside the plant cell to release effector proteins. This invokes a second line of defence in plants to produce effector-specific resistance (R) proteins encoded by *R* genes. The resulting hypersensitive reaction or effector-triggered immunity (ETI) constitutes qualitative resistance (Kushalappa and Gunnaiah, 2013). PAMP also triggers phytohormones, such as jasmonic acid, salicylic acid, ethylene, abscisic acid, and gibberellins, to provide broad biochemical resistance and reduce pathogen growth (Robert-Seilaniantz *et al.*, 2011). These phytohormones fine-tune the pathways involved in the production of secondary metabolites that impart quantitative resistance. Following recognition of PAMPs, the receptors trigger a phosphorylation cascade through the mitogen-activated protein kinase (MAPK) signalling pathway (Chisholm *et al.*, 2006), leading to the induction or repression of target genes (Czernic *et al.*, 1999). Among the induced target genes are a variety of transcription factors that regulate plant defence responses involving up- or down-regulation of resistance-related (*RR*) genes by binding to specific DNA sequences in their promoter regions (Alves *et al.*, 2014).

WRKY domain-containing proteins are one of the largest families of transcriptional regulators in plants. They have a DNA-binding domain called a W-box (TTGACC/T or WRKYGQK) and are involved in plant immune responses (Rushton *et al.*, 2010). Overexpression of WRKY transcription factor increased tolerance to pathogen infection through induction of phytohormone-mediated signalling pathways in grapes and tobacco (Dang *et al.*, 2014; Marchive *et al.*, 2013). In contrast, the overexpression of WRKY reduced resistance mechanisms in rice (Chujo *et al.*, 2013; Yokotani *et al.*, 2013) and *Capsicum* plants (Wang Y *et al.*, 2013). Although WRKY proteins might act as crucial nodes in the cross-talk between PTI and ETI, little is known regarding the molecular events occurring during the establishment of resistance or susceptibility to pathogens in plants. Also, details of molecular mechanisms of individual proteins of the WRKY superfamily are still very limited, especially in non-model plants.

The present study was designed to elucidate the mechanisms by which StWRKY1 regulates the downstream genes leading to quantitative resistance to late blight in potato based on a metabolomics approach. We identified high-fold-change RR metabolites in potato stems based on non-targeted metabolomics of two genotypes: a resistant (F06025) and a susceptible (Shepody) genotype. The two genotypes with contrasting levels of resistance to *Phytophthora infestans* induced different metabolic pathways. As reported by others, a high fold change in abundance of phenylpropanoid metabolites was observed in stems that appears to play a major role in late blight resistance by enhancing cell wall thickening through deposition of polyaromatic domains of suberin (Franke *et al.*, 2012). Phenylpropanoids were also observed in the current authors’ previous studies (Pushpa *et al.*, 2014; Yogendra *et al.*, 2014), but the molecular events in their biosynthesis and regulation were not revealed. A hierarchy of genes involved in the induction of the phenylpropanoid pathway has been identified, although this is limited to model plants. Therefore, candidate metabolites with high fold change in abundances were mapped onto metabolic pathways to identify their biosynthetic candidate genes based on genomic databases. WRKYs are implicated in the regulation of plant defence against pathogens, but only a few members of the WRKY superfamily in potato have been isolated or functionally characterized. *StWRKY1* was reported in potato following infection with *P. infestans* (Dellagi *et al.*, 2000; Machinandiarena *et al.*, 2012), but no details on metabolic pathway regulation were revealed. The current study uses a forward genetics approach to discover *StWRKY1* from the high fold change in abundance of hydroxycinnamic acid amides (HCAAs) detected based on metabolic profiling, and reverse genetics to validate in depth the transcriptional activation of *StWRKY1*, possible interactions between *StWRKY1* and HCAA biosynthetic genes, and their link to secondary cell wall strengthening to contain pathogen spread following *P. infestans* invasion.

## Materials and methods

### Plant production

A resistant genotype (F06025) and a susceptible genotype (Shepody) were obtained from Mrs Agnes Murphy (Potato Research Centre, Agriculture and Agri-Food Canada, New Brunswick, Canada). F06025 was derived from AWN86514-2×N06993-13 and Shepody was derived from Bake King×F58050. Sprouted tubers, either one piece with at least two eyes or one whole small tuber, were planted in a pot containing promix BX® and perlite. Temperature (20±3 °C with 16h photoperiod) and relative humidity (70±10%) were maintained in the greenhouse throughout the growing period.

### Pathogen production, inoculation, and incubation

*P. infestans* culture [clonal lineage US-8, A2 mating type, isolate (1661), obtained from Dr. H. Platt (AAFC, Charlottetown, Prince Edward Island, Canada)] was maintained on potato dextrose agar media. For inoculation, fresh sporangia were produced by inoculating thin potato tuber slices and incubating in Petri dishes lined with moist filter paper at 18 °C. A spore suspension was prepared by extracting sporangia in sterile water and the spore concentration was adjusted to 1×10^5^ spores ml^–1^.

Young stems of 5-week-old plants were inoculated with 20 µl sporangial suspension or mock-solution at two spots, corresponding to opposite sides of the stem, using a Hamilton syringe fitted with an autodispenser (Gastight 1750 DAD W/S; Hamilton, Reno, NV, USA). The inoculated drops were covered with Whatman filter paper discs (4–5mm diameter) to prevent spread of the spore suspension. Immediately after inoculation, the plants were covered with transparent plastic bags sprayed with sterile water on the inside to maintain high humidity to facilitate infection. The covers were removed 72h post-inoculation (hpi).

### Disease severity assessment and pathogen biomass quantification

The experiment was conducted as a randomized complete block design with two genotypes (resistant and susceptible) inoculated with *P. infestans* and three replicates over time. Each experimental unit consisted of five pots, each with two plants and five stems inoculated on opposite sides. Following inoculation, the plants were covered with plastic bags for 72h. Disease severity was assessed by measuring lesion length using a digital caliper at 3 d intervals until 9 d post-inoculation (dpi). Lesion length (mm) was used to calculate area under the disease progress curve (AUDPC). Relative biomass of *P. infestans* in the infected samples was quantified based on quantitative PCR (qPCR) (Asai *et al.*, 2008). Genomic DNA was isolated from *P. infestans*-infected stems (6 dpi) using a DNeasy Plant Mini Kit (Qiagen, Canada). qPCR was performed using IQ SYBR Green Supermix (Bio-Rad, Canada) in a CFX384^TM^ Real-Time System (Bio-Rad, Canada) according to the manufacturer’s instructions, using specific primers to amplify and detect *P. infestans* DNA and potato DNA (Supplementary Table S1 at *JXB* online).

### Sample collection, metabolite extraction, and LC-high-resolution MS (LC-HRMS) analysis

The experiment was conducted as a completely randomized block design with two genotypes (F06025 and Shepody) and two inoculations (pathogen and mock) with five replicates over time. The experimental units consisted of five plants with a total of 10 stems inoculated for each treatment. At 72 hpi, 1cm stem segments, containing the inoculated spot, were cut using a sterile scalpel. Samples were flash frozen in liquid nitrogen and stored at –80 °C. Metabolites were extracted using 60% aqueous methanol with 0.1% formic acid (De Vos *et al.*, 2007). These were analysed in negative ionization mode using a LC-HRMS system (LC-ESI-LTQ Orbitrap; Thermo Fisher, Waltham, MA, USA), with a 5cm kinetex column, as described previously (Yogendra *et al.*, 2014).

### LC-HRMS output processing

The LC-HRMS output Xcalibur RAW files were converted into mzXML format. Data was processed using the interactive LC-MS data processing software MZmine-2 with the high sensitivity peak detection algorithm XCMS centWave (Pluskal *et al.*, 2010). The observed masses and their abundance (relative intensity) were imported to MS Excel; peaks that were not consistent among replicates and those annotated as isotopes and adducts were excluded from further analyses (Yogendra *et al.*, 2014).

### Identification of RR metabolites

Monoisotopic mass peak intensity (*m*/*z*) data was subjected to pairwise Student’s *t*-test analysis in RM versus SM, RP versus RM and SP versus SM (RM, mock-inoculated resistant genotype; SM, mock-inoculated susceptible cultivar; RP, pathogen-inoculated resistant genotype; SP, pathogen-inoculated susceptible cultivar) as described previously (Kushalappa and Gunnaiah, 2013). Metabolites with significantly higher abundance in the resistant compared with the susceptible genotype were considered RR metabolites. RR metabolites were further classified into RR constitutive (RRC) and RR induced (RRI) metabolites. The RR metabolites were putatively identified based on two criteria: (i) accurate mass match (accurate mass error <5 ppm) with metabolites reported in different databases [METLIN, KNApSAcK, Plant Metabolic Network (PMN), LIPIDMAPS, and KEGG] and (ii) fragmentation pattern match with those in databases or *in silico* verification (Yogendra *et al.*, 2014).

### RNA isolation and quantitative real-time PCR (qRT-PCR)

For qRT-PCR, total RNA was isolated from stems inoculated with pathogen or mock-solution at 48 hpi in three replicates (five stems were collected from five plants for each replicate) using a RNeasy Plant Mini Kit (Qiagen, Canada). Purified RNA (3 µg from each sample) was reverse transcribed using an Affinity Script qRT-PCR cDNA Synthesis Kit (Agilent Technologies, USA). For qRT-PCR, Primer-BLAST software was used to design primer sets for *StWRKY1*, 4-coumarate:CoA ligase (*St4-CL*), tyramine hydroxycinnamoyl transferase (*StTHT*), hydroxycinnamoyl transferase (*SpHCT*), and tyrosine decarboxylase (*SpTyDC*) (Supplementary Table S1), and elongation factor-1α (*StEF1α*) and β-tubulin (*Stβ-tubulin*) were used as reference genes (Nicot *et al.*, 2005). A sample of 25ng cDNA was used in a qRT-PCR reaction using IQ SYBR Green Supermix (Bio-Rad, Canada) in a CFX384^TM^ Real-Time System (Bio-Rad) in a 10 µl reaction volume according to the manufacturer’s instructions. The relative gene expression level was calculated using the 2^–∆∆*C*T^ method (Livak and Schmittgen, 2001).

### Histochemical analysis of potato stems

Fifteen young potato internodes were collected from pathogen- and mock-inoculated plants at 72 hpi for histochemical study. Internodes were cut 0.5cm above and 0.5cm below the inoculation site using a scalpel and immediately frozen at –20 °C. For cryosectioning, tissues were embedded in Shandon Cryomatrix (Richard-Allan Scientific, USA). The cross-sectioning was carried using a cryotome (Leica, Canada) with 35 µm thickness and collected on glass slides. Sections were washed with distilled water for 2min, stained with Neu’s reagent [1% 2-amino ethyl diphenyl borinate (Sigma-Aldrich, Canada) in absolute methanol] for 5min, washed with water again for 30 s, and mounted in 15% glycerol (Alemanno *et al.*, 2003). The cross-sections of five internodes for each treatment with at least five sections from each internode were observed under a fluorescent microscope (Nikon, USA) for chemifluorescence with blue laser diode excitation at 405nm using a HQ442/45 emission filter.

### Virus-induced gene silencing (VIGS) vector construction

Tobacco rattle virus [pTRV1 (YL192) and pTRV2 (pYL154)] vectors (Liu *et al.*, 2002) were obtained from the Arabidopsis Biological Resource Center, The Ohio State University, Columbus, OH, USA. To generate pTRV2-StWRKY1, a 519bp segment of potato *WRKY1* was PCR amplified from resistant potato cDNA using primers WRKY1-F (5′-TACGTC*GAATTC*ATGGAGAATTATGCAACAATA-3′) and WRKY1-R (5′-TAGCTG*CTCGAG*TTAAAAGGAAGTA TAGATTTGCAT-3′) with *Eco*RI and *Xho*I restriction sites (underlined) at the 5′ and 3′ ends of the amplified fragment, respectively. The PCR product was digested with *Eco*RI/*Xho*I and inserted into the same site of the pTRV2 vector, resulting in pTRV2-StWRKY1. For silencing phytoene desaturase (PDS), a 396bp segment of the potato *PDS* gene was amplified from the resistant genotype and cloned into pTRV2 vector using primers (PDS-F: 5′-TACGTC*GAATTC*CAGTGGATA TTTTCAAGCTGCTT-3′; PDS-R: 5′-TAGCTG*CTCGAG*AAAC AGACCTTGGAGTTTTGACA-3′) with *Eco*RI and *Xho*I restriction sites (underlined) at the 5′ and 3′ ends of the amplified fragment, respectively. The positive clones in the pTRV2 plasmid were confirmed by sequencing. All TRV-VIGS clones were transformed into *Agrobacterium tumefaciens* strain GV3101 using a standard transformation protocol.

### *Agrobacterium*-mediated virus infiltration (agroinfiltration)

Cultures of *A. tumefaciens* strain GV3101 containing each of the constructs derived from pTRV2, with an empty vector as a control, and pTRV1 were grown at 28 °C for 24h in Luria–Bertani (LB) medium supplemented with 10mM MES, 200 μM acetosyringone, 50mg l^–1^ gentamycin, and 50mg l^–1^ kanamycin. *Agrobacterium* cells were harvested and suspended in the infiltration buffer (10mM MgCl_2_, 200 μM acetosyringone, and 10mM MES, pH 5.6). *Agrobacterium* cultures were pelleted, resuspended in infiltration buffer, and adjusted to an OD_600_ of 1.0. Mixtures of *Agrobacterium* cultures containing pTRV1 and pTRV2 and its derivatives, in a ratio of 1:1 (v/v), were placed in the dark at room temperature for 4h before infiltration. For infiltration, 0.1mm holes were made in the leaflets of 2-week-old potato plants using the corner of a new razor blade and infiltrated with *Agrobacterium* cultures (agroinfiltration) using a 1ml needleless syringe. Plants were maintained at 18 °C in a growth chamber.

### Induction of protein expression and purification of recombinant protein

Full-length cDNA of the *StWRKY1* gene was amplified by RT-PCR using *Solanum tuberosum* cDNA as template and StWRKY1-F (5′-*GGATCC*TGAGAATTATGCAACAATATTTC-3′) and StWRKY1-R (5′-*AGATCT*TTAAAAGGAAGTATAG ATTTGC-3′) primers containing *Bam*HI and *Bgl*II restriction sites (underlined). PCR product was digested with *Bam*HI and *Bgl*II, and the fragment was ligated to pTrcHis B (Invitrogen, USA) vector and transformed into *Escherichia coli* BL21 cells. *E. coli* cells harbouring *StWRKY1* were grown in 200ml LB containing 50 µg ml^–1^ ampicillin incubated at 37 °C and 250rpm. Induction of protein expression and purification was achieved using a Ni-NTA column (Qiagen, Germany) as described previously (Kumar *et al.*, 2012; Kumar *et al.*, 2014). The purified protein fractions of recombinant StWRKY1 were detected by 12% SDS-PAGE by staining with Coomassie Brilliant Blue.

### EMSA

The promoter region of *THT*, having the putative StWRKY1-binding site, was used to design synthetic single-stranded forward (5′-TCGTCAGAG*TTGACC*TCCACCAACCA-3′) and reverse (5′-TGGTTGGTGGA*GGTCAA*CTCTGACGA-3′) 26bp olig- onucleotide primers. The underlined region of the primers represents the WRKY-binding domain. Single-stranded primers were annealed at room temperature to generate a double-stranded probe, which was labelled using a Pierce Biotin 3′ End DNA Labeling Kit (Thermo Scientific, USA). EMSA was performed using a LightShift® Chemiluminescent EMSA Kit (Thermo Scientific, USA) as per the manufacturer’s instructions. Briefly, each reaction contained 2 μl 10× binding buffer, 1.0 μl each 1 µg µl^–1^ poly(dI–dC), 50% glycerol, 1% NP-40, 1M KCl, 100mM MgCl_2_, 200mM EDTA, 3 µl (3 μg) purified protein, and 2 µl (20fmol) labelled probe or unlabelled DNA as the competitor made up to a final volume of 20 µl. The binding reactions were incubated at 25 °C for 20min. A 6% polyacrylamide minigel (containing 3% glycerol) was pre-run for 30min using 0.5× TBE as a running buffer. Then 5 μl 5× loading buffer was added to the reaction and a 25 μl aliquot of the reaction mixture was subjected to electrophoresis. The gel was transferred to a nitrocellulose membrane (Amersham Biosciences, USA) using a Mini Trans-Blot® Electrophoretic Transfer Cell (Bio-Rad, USA), followed by cross-linking of the membrane below a UV lamp. The biotin-labelled DNA was detected using a Chemiluminescent Nucleic Acid Detection Module (Thermo Scientific, USA) as per the manufacturer’s instructions. The change in band position representing different molecular weights was used to confirm the shift.

### Bimolecular fluorescence complementation (BiFC) assay

For the BiFC assay, sequences encoding *StWRKY1* and *StMEK1* were amplified and fused with the N- and C-terminal parts of Yellow Fluorescent Protein (YFP^n^ and YFP^c^, respectively). BiFC plasmids containing YFP^n^ and YFP^c^ fusions were co-transformed into potato protoplasts as described previously (Walter *et al.*, 2004). The protoplasts were incubated under weak light for 12–16h before observation. YFP fluorescence signals were checked under a fluorescent microscope (Nikon, USA). The presence of yellow fluorescence was used to discriminate treatments.

### Luciferase (LUC) transient expression assay

For LUC transient expression assays, reporter plasmids (4-CLp-LUC or THTp-LUC), effector constructs containing *StWRKY1*, and 35S::β-glucuronidase (GUS) internal control were co-transformed into potato protoplasts. The protoplasts were pelleted and resuspended in 1× cell culture lysis reagent (Promega, USA). GUS fluorescence was measured using a Modulus luminometer/fluorometer with a UV fluorescence optical kit (Fluorescence Microplate Reader; BioTek, USA). The experiment was carried out in three replicates; each replicate contained 20 µl protoplast lysate and 100 µl LUC mix. LUC activity was detected with a luminescence kit using LUC assay substrate (Fluorescence Microplate Reader). The relative reporter gene expression levels were expressed as LUC/GUS ratios, which were used to discriminate treatments.

## Results

### Late blight severity and *P. infestans* biomass

**Fig. 1.**
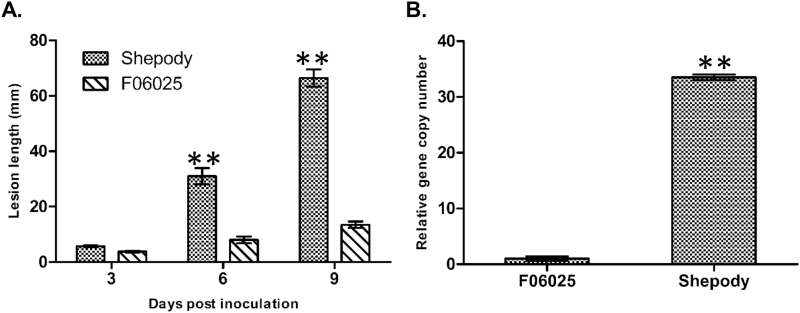
Disease progress curve and pathogen biomass in infected stems of resistant (F06025) and susceptible (Shepody) genotypes inoculated with *P. infestans:* (A) Late blight disease severity progress curve based on lesion length (mm) and (B) biomass of pathogen quantified as relative *P. infestans*-specific (O-8) gene expression at 6 dpi. Significant differences between susceptible and resistant plants using Student’s *t*-test: *P<0.05; **P<0.01.

Late blight severity in the potato genotypes F06025 and Shepody was assessed by measuring lesion length over time following inoculation with *P. infestans* clonal lineage US-8. The initial symptoms were observed as brown-coloured lesions on the inoculated stems at 3 dpi in both F06025 and Shepody. By 9 dpi, the stem lesion length was 66.44mm (AUDPC=201.09) in Shepody, differing (*P*<0.001) from the resistant genotype F06025 with a lesion length of 13.44mm (AUDPC=49.71) (Fig. 1A and Supplementary Fig. S1 at *JXB* online).

Pathogen biomass in the infected stems of genotypes F06025 and Shepody were quantified based on qPCR. At 6 dpi, *P. infestans*-specific gene copy numbers (DNA, O-8) were 33.52-fold higher (*P*<0.001) in the susceptible Shepody than in the resistant F06025 genotype (Fig. 1B).

### Differential accumulation of metabolites in stems of potato genotypes

Metabolites were profiled in the stems of resistant and susceptible genotypes at 3 dpi following inoculation with *P. infestans* or with water and analysed based on LC-HRMS. A total of 8408 consistent peaks of monoisotopic masses were detected in all the replicate and treatment combinations. Among the mock-inoculated plants, a total of 679 metabolites had higher abundance in the resistant than in the susceptible genotype, and these were designated as RRC (RM>SM) metabolites (Table 1, and Supplementary Fig. S2A and Supplementary Table S2 at *JXB* online). These metabolites belonged to different chemical groups (Supplementary Fig. S2B) and some important metabolites had high fold changes in the resistant genotype [phenylpropanoids: 1,2-di-*O*-sinapoyl-β-d-glucose (14.55), 1-*O*-sinapoyl-β-d-glucose (6.31) and scopolin (3.15); glycerophospholipids: 1-(9*Z*-nonadecenoyl)-glycero-3-phosphoserine (3.61), 1-hexadecyl-2-propionyl-*sn*-glycero-3-phosphocholine (3.50) and 1-octadecyl-2-ethyl-*sn*-glycero-3-phosphocholine (3.14); sphingolipids: (3′-sulfo) Galβ-Cer (d18:0/20:0(2OH)) (3.77), *N*-palmitoylsphingosine (2.90) and *N*-(eicosanoyl)-sphinganine (2.37)].

**Table 1.** *Fold change in abundance of RR metabolites detected in potato stem following* P. infestans *or mock-solution inoculation*

Observed mass (Da)	Exact mass (Da)	Name	Fold change
*Phenylpropanoids*			
148.0528	148.0524	*trans*-Cinnamic acid	1.24* RRI
162.032	162.0317	4-Hydroxycoumarin	1.36* RRC
165.0793	165.0790	l-Phenylalanine	1.18* RRI
166.0633	166.0630	Dihydro-3-coumaric acid	2.13** RRI
174.0531	174.0528	Shikimate	1.31* RRI
181.0742	181.0739	α-Amino oxy-β-phenyl propionate	8.22* RRI; 1.74* RRC
192.0273	192.0270	Citrate	2.27* RRI
192.0637	192.0634	l-Quinic acid	2.46* RRC; 1.18* RRI
194.0586	194.0579	Ferulic acid	12.44** RRC; 3.04** RRI
208.0552	208.0558	Methyl 5-(1-propynyl)-2-thiophenepropanoate	2.95** RRC
264.1472	264.1474	Feruloylputrescine	5.03** RRI
300.0844	300.0845	Salicylate β-d-glucose ester	1.59** RRI
306.1690	306.1692	Feruloylagmatine	5.01** RRI; 3.11** RRC
313.1313	313.1314	*N*-feruloyltyramine	12.85** RRI; 1.99** RRC
329.1262	329.1263	*N*-feruloyloctopamine	8.90** RRI
342.0946	342.0951	Caffeic acid 3-glucoside	2.72* RRC
354.0950	354.0951	Scopolin	3.15* RRC; 1.11* RRI
368.1103	368.1107	5-*O*-feruloylquinic acid	2.63* RRC
386.1210	386.1213	1-*O*-sinapoyl-β-d-glucose	6.31** RRC; 2.92* RRI
537.1823	537.1846	4-Demethylsimmondsin 2′-(*E*)-ferulate	2.98* RRC
582.1941	582.1949	10-Acetoxyligustroside	2.74* RRC
592.1790	592.1792	1,2-Di-*O*-sinapoyl-β-d-glucose	14.55** RRC; 1.13* RRI
*Sphingolipids*			
479.4357	479.4338	Cer(d14:2(4*E*,6*E*)/16:0)	1.30* RRI
537.5138	537.5121	Cer(d18:1/16:0)	2.90* RRC; 1.44* RRI
595.5880	595.5903	Cer(d18:0/20:0)	2.37* RRC; 1.15* RRI
683.5331	683.5336	GlcCer(d15:2(4*E*,6*E*)/18:0)	1.30* RRC
711.5643	711.5649	GlcCer(d15:2(4*E*,6*E*)/20:0)	1.24* RRI
851.5776	851.5793	(3′-Sulfo)Galβ-Cer(d18:0/20:0(2OH))	3.77** RRC
*Glycerophospholipids*			
537.309	537.3067	1-(9*Z*-Nonadecenoyl)-glycero-3-phosphoserine	3.61* RRI
537.379	537.3794	1-Hexadecyl-2-propionyl-*sn*-glycero-3-phosphocholine	3.50*RRI
537.4151	537.4158	1-Octadecyl-2-ethyl-*sn*-glycero-3-phosphocholine	3.14* RRI

Detailed compound identification is presented in Supplementary Table S2. Fold change calculation was based on relative intensity of metabolites: RRC=RM/SM and RRI=(RP/RM)/(SP/SM) (see main text for details). Significance (*t*-test): *P<0.05, **P<0.01.

Among the pathogen-inoculated plants, 167 metabolites were induced with greater abundances in the resistant than in the susceptible genotype, and these were designated as RRI [(RP>RM)>(SP>SM)] metabolites (Supplementary Fig. S2A and Supplementary Table S2). Out of 167 RRI metabolites, 97 were putatively identified (Table 1 and Supplementary Table S2); the identity of the most significant metabolites was confirmed based on fragmentation patterns using LC-HRMS (Supplementary Fig. S3 at *JXB* online). Thirty-four of the identified metabolites were phenylpropanoids, including the HCAAs *N*-feruloyltyramine, *N*-feruloyloctopamine, feruloylputrescine, and feruloylagmatine. In addition to phenylpropanoids, 24 fatty acids and four alkaloids were induced in higher abundance in the resistant than in the susceptible genotype following pathogen inoculation.

**Fig. 2.**
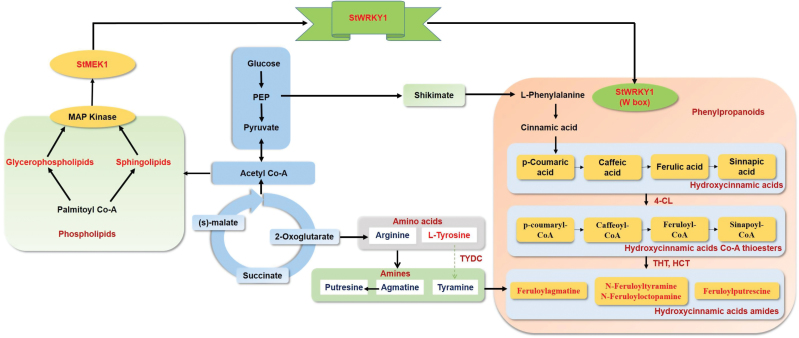
Satellite metabolic pathway of potato–*Phytophthora* interaction showing the RR metabolites and their catalysing enzymes involved in their biosynthesis detected in resistant potato inoculated with *P. infestans* or mock-solution. Metabolites and genes detected in this study are marked in red. Detailed interactions are presented in Fig. 7. (This figure is available in colour at *JXB* online.)

Among the RRI metabolites identified, most of the high-fold-change metabolites belonged to the phenylpropanoid pathway, including the HCAAs *N*-feruloyltyramine (12.85), *N*-feruloyloctopamine (8.90), feruloylputrescine (5.03), and feruloylagmatine (5.01), which are known to be involved in cell wall thickening. To confirm the deposition of HCAAs in cell walls, a histochemical staining technique was used to visualize the location of deposition of these metabolites. Deposition of HCAAs (blue fluorescence intensity) was greater in pathogen-treated resistant than in mock-treated resistant and pathogen- or mock-treated susceptible genotypes (Supplementary Fig. S4 at *JXB* online). The candidate RRI metabolites with the highest fold changes in abundance following pathogen inoculation were mapped onto their metabolic pathways (Fig. 2) to identify the catalytic enzymes, which were then searched in the Spud database (http://solanaceae.plantbiology.msu.edu/; accessed on 25 February 2015) to identify candidate *RR* genes. The *RR* genes that encoded the enzymes of these RRI metabolites were 4-coumarate:CoA ligase (*4-CL*) (GenBank accession number AF150686.1; chromosome 3), hydroxycinnamoyl transferase (*HCT*) (Gene ID: PGSC0003DMT400036695; chromosome 3), tyramine hydroxycinnamoyl transferase (*THT*) (GenBank accession number JX896425.1; chromosome 10), and tyrosine decarboxylase (*TyDC*) (Gene ID: PGSC0003DMT400038501; chromosome 3) (Fig. 2). The expression of *RR* genes is transcriptionally regulated by the co-ordinated action of transcription factors which bind to *cis*-acting elements and regulate the expression of *RR* genes. Previous studies reported that *StWRKY1* (GenBank accession number AJ278507.1; chromosome 5) is induced and co-regulated with class I endochitinase during the compatible interaction of potato and *P. infestans* (Dellagi *et al.*, 2000). The promoter regions of *RR* genes were sequenced and the presence of the W1-box of the WRKY1 transcription factor was confirmed (Supplementary Table S3 at *JXB* online).

### Metabolic regulation of phenylpropanoid pathway genes by *StWRKY1*

**Fig. 3.**
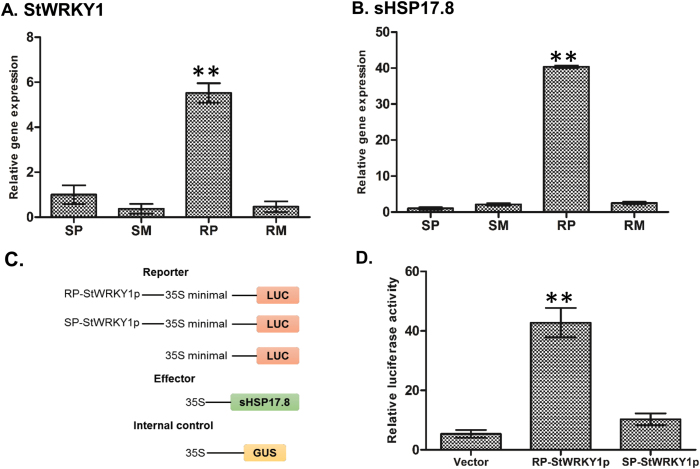
Relative transcript expression and regulation of *StWRKY1* by *sHSP17.8*. The relative transcript expression for (A) *StWRKY1* and (B) *sHSP17.8* was confirmed in the resistant genotype relative to the susceptible genotype following *P. infestans* and mock-solution inoculation at 48 hpi using qRT-PCR in comparison with reference genes for elongation factor-1α and β-tubulin. See main text for further details. Regulation of *StWRKY1* by *sHSP17.8* was confirmed by LUC transient expression assay: (C) constructs used and (D) relative LUC reporter activity by HSP17.8. Values are averages of three replicates. Significant differences in relative expression levels using Student’s *t*-test: *P<0.05; **P<0.01.

To investigate whether *StWRKY1* is involved in defence responses, *StWRKY1* transcripts were quantified based on qRT-PCR. The relative transcript abundance of *StWRKY1* was higher (*P*<0.001) in resistant (5.52) than in susceptible genotypes (1.00) (Fig. 3A). Furthermore, the coding and promoter regions of *StWRKY1* were amplified from both resistant and susceptible genotypes, and cloned into the pGEM®-T Easy vector and verified by Sanger sequencing. No sequence variation was observed for the coding region of *StWRKY1* in both resistant and susceptible genotypes. However, the heat shock element (HSE) domain was detected in the promoter region of the resistant genotype and was completely absent in the susceptible genotype (Supplementary Fig. S5 and Supplementary Table S4 at *JXB* online). HSEs act as heat shock protein (HSP)-binding sites and help to induce hypersensitive responses by generating oxidative bursts.

### Transcriptional regulation of *StWRKY1* by small HSPs (sHSPs)

RNA sequencing was carried out between the resistant genotype F06025 and another susceptible genotype, Russet Burbank, using 48 hpi potato leaf samples. Sequencing was performed with an Illumina HiSeq 2000 sequencer with 100bp paired-end reads (Genome Québec-McGill University Innovation Center, Montreal, Canada). The reads were aligned to the potato reference genome (Potato Genome Sequencing Consortium, 2011). We observed a high fold change in expression for *sHSP17.8* (77.78) and *sHSP26.5* (9.00) in the resistant genotype (F06025), compared with the susceptible genotype (Russet Burbank) upon *P. infestans* infection, based on RNA sequencing analysis (unpublished data and Supplementary Table S5). The high fold change in expression of *sHSP17.8* was further validated using qRT-PCR. Interestingly, qRT-PCR revealed a higher fold change of 40.35 transcripts in the resistant (F06025) than in the susceptible (Shepody) genotype (Fig. 3B). As HSPs are transcriptionally activated by heat shock factors (HSF) to interact with HSEs present on the promoter region of a gene, the possible regulation between *sHSP17.8* and HSE was tested by *in vivo* LUC assay in potato protoplasts (Fig. 3C, D). The *StWRKY1* promoter from the resistant genotype had a relative LUC value of 42.77 as compared (*P*<0.001) with the susceptible genotype (10.25) and control vector (5.38). Taken together, these results suggest that *sHSP17.8* interacts with the HSE conserved domain present in the promoter region of *StWRKY1* in the resistant genotype and is likely involved in mediating the high transcriptional activity of StWRKY1 proteins.

### Activation of transcription factor StWRKY1 by MAPK

**Fig. 4.**
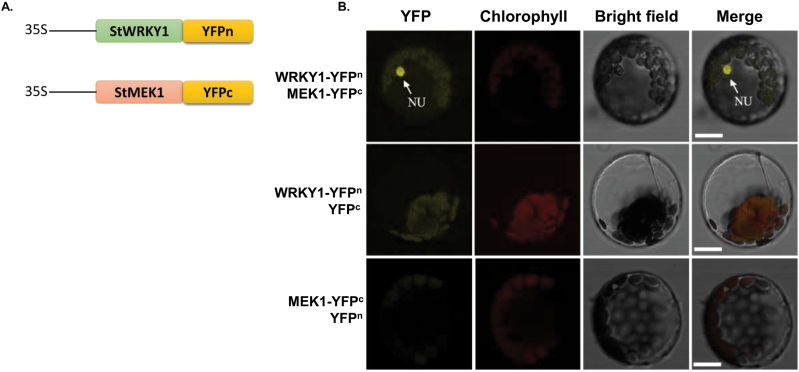
BiFC assay for StWRKY1 and StMEK1 interaction: (A) constructs used in the BiFC expression assay, and (B) BiFC assay showing the interaction between StWRKY1-YFP^c^ and YFP^n^-StMEK1. The nucleus (NU) is shown in yellow and chlorophyll autofluorescence is shown in red. Bars, 5mm. (This figure is available in colour at *JXB* online.)

We detected a high fold change in abundance of induced phospholipid metabolites in the resistant genotype following pathogen infection [sphingolipids: (3′-sulfo)Galβ-Cer(d18:0/20:0(2OH)) (3.77), *N*-palmitoylsphingosine (2.90), and *N*-(eicosanoyl)-sphinganine (2.37); glycerophospholipids: 1-(9*Z*-nonadecenoyl)-glycero-3-phosphoserine (3.61), 1-hexadecyl-2-propionyl-*sn*-glycero-3-phosphocholine (3.50), and 1-octadecyl-2-ethyl-*sn*-glycero-3-phosphocholine (3.14)]. These phospholipids are structural components of membranes and act as novel secondary messengers in plant defence signalling pathways. These activate the MAPK cascade by triggering oxidative bursts (Laxalt and Munnik, 2002). MAPK (StMEK1) phosphorylates StWRKY1, which in turn activates defence gene expression (Katou *et al.*, 2003). To substantiate the interaction between StWRKY1 and StMEK1 in potato cells, a BiFC assay was performed in which YFP^n^ fused to StWRKY1 (YFP^n^-StWRKY1) and YFP^c^ fused to StMEK1 (StMEK1-YFP^c^) were transiently co-expressed in potato protoplasts (Fig. 4A). Co-expression of YFP^n^-StWRKY1 and StMEK1-YFP^c^ reconstituted a functional YFP in the nucleus, whereas co-expression with either control vector failed to generate YFP fluorescence, indicating StMEK1 is required for activation of StWRKY1 (Fig. 4B).

### StWRKY1 physically interacts with promoters of HCAA biosynthetic genes and exhibits transcriptional activity

**Fig. 5.**
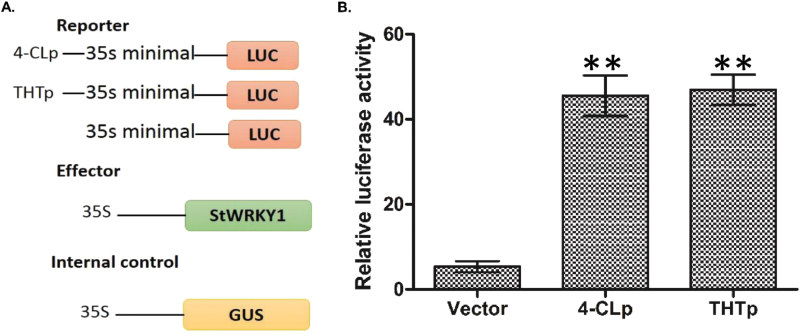
Transcriptional regulation of HCAA biosynthetic genes by StWRKY1: (A) constructs used in the transient expression assay and (B) relative LUC reporter activity by StWRKY1. Values are averages of three replicates. Significant differences in expression levels in promoters compared with vector using Student’s *t*-test: *P<0.05; **P<0.01.

To investigate whether the StWRKY1 proteins are involved in transcriptional regulation of HCAA biosynthesis, the coding region of the *StWRKY1* gene and promoters of *4-CL* and *THT* from the resistant genotype were amplified and cloned into pGEM®-T Easy vector (Promega, USA), as confirmed by sequencing. This was followed by subcloning into the FU63 (CD3-1841) (Wang X *et al.*, 2013) vector with a LUC gene driven by the 35S minimal promoter (Fig. 5A). The constructs were transformed into potato protoplasts. We found that StWRKY1 bound to the promoter region of *RR* genes, and drastically activated LUC reporter expression in *4-CL* (45.55) and *THT* (47.00) promoters compared with vector alone (4.16) (Fig. 5B), suggesting the transcriptional regulation of HCAA biosynthesis.

The HCAAs, including *N*-feruloyltyramine, *N*-feruloyloctopamine, feruloylagmatine, and feruloylputrescine, play a critical role in secondary cell wall thickening (Gunnaiah *et al.*, 2012; Pushpa *et al.*, 2014; Yogendra *et al.*, 2015; Yogendra *et al.*, 2014). Accordingly, the physical interaction of StWRKY1 with HCAA biosynthetic gene *THT* was tested by *in vitro* EMSA as additional evidence (Supplementary Fig. S6 at *JXB* online). For EMSA, the purified recombinant StWRKY1 protein fractions had a molecular weight of 25kDa (Supplementary Fig. S6A) as analysed on 12% SDS-PAGE. This was in agreement with the theoretical molecular weight of 23kDa (19.86kDa for the native protein plus 3kDa for the His-tag). The purified recombinant protein was used to study the interaction with the W1-box of the *THT* promoter. EMSA results showed a shift in signal of biotinylated *THT* promoter that was inhibited in the presence of excess unlabelled competitor (Supplementary Fig. S6B). These results indicate that StWRKY1 can specifically bind to the W1-box of the *THT* promoter and can regulate its gene expression.

### Functional validation of StWRKY1

The loss-of-function experiment was performed with *StWRKY1*-silenced potato seedlings to elucidate the biological function of *StWRKY1* in late blight resistance mechanisms. Nucleotide sequences of 519bp of *StWRKY1* and 396bp of *StPDS* (GenBank accession number AY484445.1) were amplified from the resistant potato genotype F06025 and cloned into the pTRV2 vector to develop the TRV-VIGS construct (Supplementary Fig. S7A at *JXB* online). A mixture of *Agrobacterium* cultures containing pTRV1 and pTRV2-StWRKY1 or pTRV2-StPDS was infiltrated onto 2-week-old resistant potato seedlings. Agroinfiltrated plants were healthy and no plant died from this treatment, although stunted growth was observed. Plants expressing pTRV2-StPDS showed photobleaching after 30 d of agroinfiltration and used as positive controls to identify plants that were successfully silenced (Supplementary Fig. S7B). The *PDS* gene was used to demonstrate the effectiveness of VIGS, as this gene participates in the carotenoid metabolic pathway. *StWRKY1*-silenced leaves exhibited higher levels of *P. infestans* susceptibility compared with non-silenced plants. At this phase, *P. infestans* spores (~1000 sporangia) were inoculated to stems of *StWRKY1*-silenced plants, and transcript expression levels, metabolites, and pathogen biomasses were used to validate the resistance function.

**Fig. 6.**
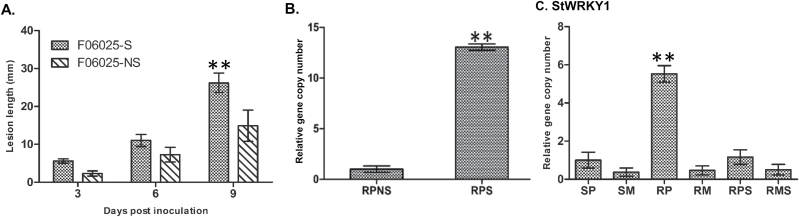

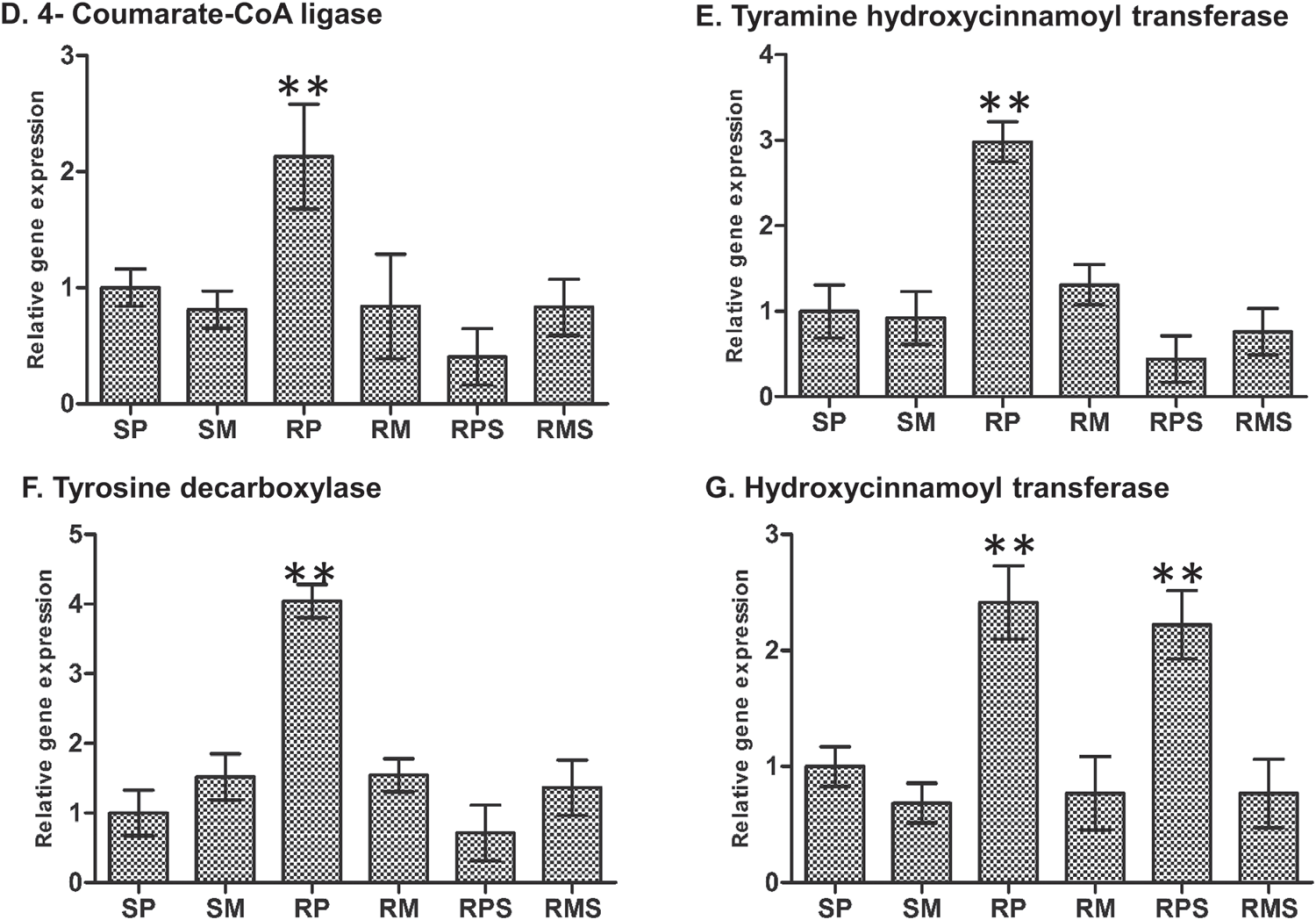
Effect of *StWRKY1* silencing on late blight resistance mechanisms. (A) Late blight disease severity progress. Disease severity was quantified as lesion length (mm) at 3 d intervals. (B) Biomass of *P. infestans* on silenced (S) and non-silenced (NS) resistant potato genotypes. Biomass was quantified as relative *P. infestans*-specific (O-8) gene expression at 6 dpi. Relative transcript expression of (C) StWRKY1, and phenylpropanoid genes for (D) 4-coumarate:CoA ligase, (E) tyramine hydroxycinnamoyl transferase, (F) tyrosine decarboxylase, and (G) hydroxycinnamoyl transferase. Relative transcript expression was measured at 48 hpi in comparison with reference genes for elongation factor-1α and β-tubulin. RPS indicates resistant silenced and inoculated with *P. infestans*; RMS indicates resistant silenced and inoculated with mock-solution; see main text for further details. Significant differences in expression levels in RP compared with SP using Student’s *t*-test: *P<0.05, **P<0.01.

To assess the effect of *StWRKY1* in late blight resistance, the disease severity and biomass were quantified in silenced and non-silenced resistant plants. The lesion length was higher (*P*<0.001) in silenced plants (26.19mm; AUDPC=80.68), significantly differing from the non-silenced plants (14.90mm; AUDPC=47.60). Likewise, pathogen biomass was higher (*P*<0.001) in silenced plants (fold change=13.06) compared with non-silenced plants at 6 dpi (Fig. 6A, B). Furthermore, the relative abundances of the transcripts of phenylpropanoid genes (*4-CL*, *THT*, *TyDC*, and *HCT*) were determined in silenced and non-silenced resistant plants with reference to the susceptible genotype (Fig. 6C–F). *StWRKY1* and the regulated *RR* genes (*4-CL*, *THT* and *TyDC*) were reduced (*P*<0.001) in silenced plants (fold change=1.16, 0.40, 0.44, and 0.71, respectively) as compared with resistant plants (5.52, 2.13, 2.98, and 4.04). However, no significant reduction was observed in *HCT* transcripts in silenced plants (fold change=2.22) compared with resistant plants (2.41) and this was probably due to the absence of the W1-box in the *HCT* promoter (Fig. 6G and Supplementary Table S3). We then tested the effect of *StWRKY1* silencing on the production of HCAAs. The RRI metabolites *N*-feruloyltyramine (fold change=10.13), *N*-feruloyloctopamine (6.65), feruloylputrescine (3.80), and feruloylagmatine (3.44) were reduced (*P*<0.05) after silencing (Table 2). Taken together, these results provide compelling evidence that *StWRKY1* is a positive regulator of late blight resistance through the reinforcement of secondary cell walls.

**Table 2.** *Effect of* StWRKY*1 silencing on RR phenylpropanoid metabolites upon* P. infestans *or mock-solution inoculation*

Observed mass (Da)	Exact mass (Da)	Name	Fold change before silencing	Fold change after silencing
264.1472	264.1474	Feruloylputrescine	5.03** RRI	3.80* RRI
306.1690	306.1692	Feruloylagmatine	5.01** RRI; 3.11** RRC	3.44* RRI
313.1313	313.1314	*N*-feruloyltyramine	12.85** RRI; 1.99** RRC	10.13** RRI; 1.70** RRC
329.1262	329.1263	*N*-feruloyloctopamine	8.90** RRI	6.65** RRI; 2.67* RRC

Fold change calculation was based on relative intensity of metabolites: RRC=RM/SM and RRI=(RP/RM)/(SP/SM) (see main text for details). Significance (*t*-test): *P<0.05, **P<0.01.

## Discussion

In the present study, an integrated metabolomics, gene expression, and gene sequencing information approach was used to discover and explore the role of transcription factor *StWRKY1*, and also to explain the late blight resistance mechanisms in potato genotypes F06025 and Shepody. These genotypes varied in their resistance levels and also in accumulation of metabolites. Most of the high-fold-change metabolites identified in the resistant genotype belonged to the phenylpropanoid pathway, especially HCAAs. These metabolites are well known for their role in cell wall thickening, forming antimicrobial barriers (Dangl *et al.*, 2013). Plants have developed a system to maintain cell wall integrity by activation of signalling molecules, transcriptional reprogramming and biosynthesis of defence metabolites that limit pathogen infection. Cell wall thickening reduces the spread of pathogens in plants beyond the site of infection, reducing lesion expansion. These complex molecules are not generally broken down by plant pathogens. Here, a forward genetics approach was followed to explore the upstream genes involved in the transcriptional regulation of the HCAAs biosynthetic pathway genes in potato stem, mainly through functional elucidation of *StWRKY1.*

### Induction of StWRKY1 in potato against *P. infestans* infection

During potato–*P. infestans* interaction, significantly higher fold expression of the *StWRKY1* gene was identified in the resistant genotype (fold change=5.52) as compared with the susceptible genotype (1.00). Despite the existence of numerous links between WRKY transcription factors and plant defence genes, direct evidence for WRKY proteins binding with *RR* genes and further accumulation of metabolites remains limited. Several studies have focused on model plants such as *Arabidopsis* and tobacco, but only a few focused on potato WRKYs. In potato, *StWRKY1* is strongly induced following a compatible interaction with *P. infestans* (Dellagi *et al.*, 2000) and treatment with potassium phosphite during infection inhibited pathogen spread (Machinandiarena *et al.*, 2012). Overexpression of *VvWRKY1* in grapevines provided higher resistance to downy mildew *Plasmopara viticola* through higher expression of genes involved in the jasmonic acid pathway (Marchive *et al.*, 2013). Likewise, the overexpression of *CaWRKY27* in tobacco plants was associated with resistance to *Ralstonia solanacearum* infection through induction of jasmonic acid-, salicylic acid-, and ethylene-mediated signalling pathways (Dang *et al.*, 2014). Resistance to poplar canker was associated with overexpression of *PtoWRKY60*, which regulated high expression of defence-associated genes, such as PR5.1, PR5.2, PR5.4, PR5.5, and CPR5, in poplar (Ye *et al.*, 2014). Two *CaWRKY1* genes, *CaWRKY1a* and *CaWRKY1b* (which bind to the pW1a promoter region), were identified in *Coffea arabica* and conferred resistance to the rust fungus *Hemileia vastatrix* (Petitot *et al.*, 2013). These findings depict the involvement of the WRKY transcription factors in disease resistance, as described here in potato against *P. infestans*.

We also looked into the signalling pathways which involved induction of the WRKY transcription factor and detected high fold changes in several induced phospholipid metabolites in the resistant genotype. These phospholipids act as novel secondary messengers in plant defence signalling pathways and activate the MAPK cascade by triggering oxidative bursts (Laxalt and Munnik, 2002). MAPK converts the signals generated from receptors to cellular responses. Upon activation, MAPK translocates into the nucleus and phosphorylates the WRKY transcription factor, which in turn activates downstream gene expression (Kim and Zhang, 2004). In potato, *StMEK1* was functionally characterised during defence reactions (Katou *et al.*, 2003), which are also associated with *StWRKY1*, as was confirmed here by BiFC assay. *StMEK1* and *StWRKY1* function together to induce the expression of downstream *RR* genes involved in plant defence.

### Transcriptional activation of *StWRKY1* by sHSPs only in the resistant potato genotype

We detected significantly high fold changes of *sHSP17.8* (fold change=40.35) in a resistant genotype of potato against late blight infection. The presence of HSEs on the promoter region of the *StWRKY1* gene in the resistant genotype, but not in the susceptible genotype (Supplementary Table S4), helped the sHSPs to interact and induce the transcription factor StWRKY1. Small HSPs interact and provide stability to the proteins by acting as molecular chaperones (Haslbeck and Vierling, 2015). LUC assay confirmed the transcriptional regulation of *StWRKY1* by *sHSP17.8*. The HSEs are highly conserved and consist of inverted repeats of the pentameric sequence nGAAn (Amin *et al.*, 1988). The HSE present in the promoter region of the *Arabidopsis APX1* gene acts as a functional component that helps to protect plants from oxidative stress (Storozhenko *et al.*, 1998). In tobacco, the sHSP Ntshsp17 acts as a molecular chaperone and helps in the maintenance of cellular conditions suitable for inducing defence responses against bacterial wilt pathogen *Ralstonia solanacearum* by stabilizing signalling-related proteins (Maimbo *et al.*, 2007). In tomato, the sHSP RSI2 is a HSP20 member that specifically interacts and provides stability to the *Fusarium oxysporum* R protein I-2 (Van Ooijen *et al.*, 2010). Likewise, the induction of mitochondrial HSP22 occurred in tomato during oxidative stress and helped to provide adoptive responses (Banzet *et al.*, 1998). Conflicting with these results, wheat sHSP, Mds1 present on chromosome 3AS increases susceptibility to wheat gall midge and powdery mildew fungus *Blumeria graminis* f. sp. *tritici* (Liu *et al.*, 2013).

### Transcriptional regulation of phenylpropanoid pathway *RRI* genes by StWRKY1

To combat pathogen infection, plants have developed specific metabolic pathways to biosynthesize metabolites that reinforce secondary cell walls as mechanical barriers. Following pathogen inoculation, genes involved in the production of tyramine-derived HCAAs were *4-CL*, *THT*, and *TyDC*, which were upregulated 2.13-, 2.98-, and 4.04-fold, respectively, in the resistant genotype. Concurrently, metabolites produced from these candidate *RRI* genes, i.e. *N*-feruloyltyramine and *N*-feruloyloctopamine, were induced with significantly high fold changes in the resistant genotype. The authors previous studies reported high-fold-change expression of *THT* and *TyDC* genes, along with high-fold-change accumulation of RRI metabolites *N*-feruloyltyramine, *N*-caffeoyltyramine, and N-feruloyloctopamine in leaves of resistant genotypes (Pushpa *et al.*, 2014; Yogendra *et al.*, 2014; Yogendra *et al.*, 2015). However, here, for the first time, *StWRKY1* was associated with the promoters of downstream HCAA biosynthetic genes *4-CL* and *THT in vivo* and *in vitro*. The results of the EMSA and LUC assays confirmed that *StWRKY1* physically interacts with and induces high-fold-change expression of downstream genes involved in secondary cell wall thickening. Likewise, the overexpression of *VvWRKY1* induced a cinnamyl alcohol dehydrogenase and a caffeic acid *O*-methyl transferase gene in grape vines, providing higher resistance to downy mildew (Marchive *et al.*, 2013). Taken together, these findings suggest that *StWRKY1* takes part in a regulatory network, directly controlling secondary cell wall thickening by acting on promoters of HCAA biosynthetic genes.

### Silencing of *StWRKY1* results in profound effects on secondary cell wall thickening

To further assess the effect of StWRKY1 on the resistance mechanisms, *StWRKY1* was silenced in a resistant genotype, and silenced and non-silenced resistant plants were compared based on relative gene expression of HCAA biosynthetic genes, accumulation of their downstream biosynthesized RRI metabolites, amount of fungal biomass, and disease severity. The transcript abundance of *StWRKY1* and its regulating *RRI* genes, *4-CL*, *THT*, and *TyDC*, was reduced (*P*<0.05) in silenced plants (fold change=1.16, 0.40, 0.44, and 0.71, respectively) compared with resistant plants (5.52, 2.13, 2.98, and 4.04, respectively). However, no significant reduction was observed for *HCT* transcripts, probably due to the absence of the W1-box. The abundance of the HCAA metabolites *N*-feruloyltyramine and *N*-feruloyloctopamine was reduced 2-fold after silencing, resulting in a higher (*P*<0.001) biomass in silenced plants (fold change=13.06). This may be due to the metabolic profiling protocol, where metabolite extraction, detection, and identification were not comprehensive, and thus it is possible that other *RR* genes and associated metabolites are also involved in imparting resistance to late blight. Correspondingly, silencing the hydroxycinnamoyl-CoA:hydroxycinnamoyl transferase affected cell wall strength by decreasing the dimethoxylated syringyl units of the lignin polymer in *Nicotiana benthamiana* stems (Hoffmann *et al.*, 2004). Silencing of cellulose synthase genes affected cell wall formation in flax (Chantreau *et al.*, 2015). Likewise, silencing of ethylene response factor Pti5, which is involved in ethylene signalling, increased the susceptibility to aphids of a near-isogenic genotype that carried the Mi-1.2 *R* gene in tomato (Wu *et al.*, 2015). These findings, together with the data reported here provide insights into the effect of silencing of *StWRKY1* on downstream HCAA biosynthetic genes crucial for secondary cell wall thickening in potato.

### Proposed model for the role of StWRKY1 in potato secondary cell wall thickening

**Fig. 7.**
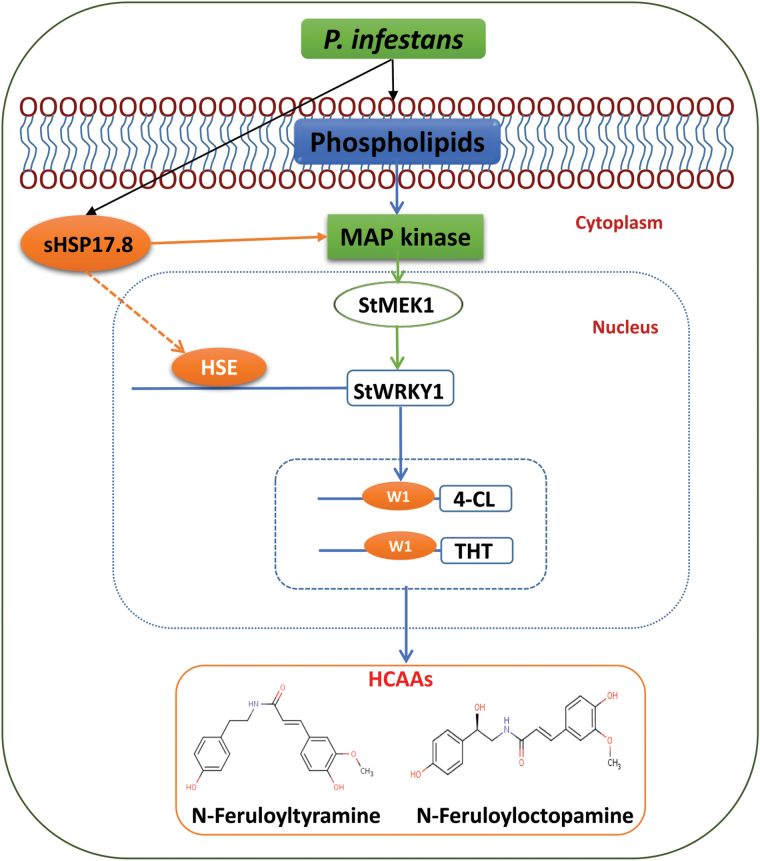
Proposed model of *StWRKY1* in regulating secondary cell wall thickening. *MEK1*, MAPK1; see main text for further details.

Based on integrated metabolomics and genomics data, the authors propose a model to explain the role of StWRKY1, together with HCAA biosynthetic genes, in mediating potato secondary cell wall thickening (Fig. 7). Following *P. infestans* invasion, a hierarchy of genes is induced in the phenylpropanoid pathway starting from the induction of phospholipid metabolites (glycerophospholipids and sphingolipids), possibly by enzymes produced by the pathogen, activating MAPK (StMEK1), which in turn phosphorylates the transcription factor (StWRKY1). Concurrently, sHSP17.8 is also induced in the resistant genotype. sHSP17.8 interacts with HSEs present in the *StWRKY1* promoter region to enable functioning of *StWRKY1*. Consequently, StWRKY1 protein interacts with HCAA biosynthetic genes *4-CL*, *TyDC*, *THT*, and possibly others, inducing high-fold-change production of the HCAA metabolites *N*-feruloyltyramine, *N*-feruloyloctopamine, and others. Taken together, these results suggest that *StWRKY1* is required for the induction of HCAAs and defence by secondary cell wall thickening in potato against *P. infestans*. These candidate genes can be used to replace non-functional genes in susceptible commercial cultivars, e.g. Shepody and Russet Burbank, based on genome-editing technologies to enhance quantitative resistance (Shan *et al.*, 2014).

## Supplementary Data

Supplementary data are available at *JXB* online.

Figure S1. *P. infestans*-infected potato stems with susceptible (Shepody) and resistant (F06025) genotypes at 9 dpi.

Figure S2. (A) The number of RR metabolites identified in stems of the resistant potato genotype inoculated with water or *P. infestans*. (B) RR metabolites identified in the resistant potato genotype inoculated with a mock-solution or *P. infestans* and their classification according to chemical groups.

Figure S3. *In silico* fragmentation of RR metabolites.

Figure S4. Laser scanning confocal micrographs of stem sections exhibiting secondary cell wall thickening due to HCAAs (HCAA blue fluorescence).

Figure S5. Comparison of sequence variation between resistant and susceptible *StWRKY1* promoter regions.

Figure S6. (A) Analysis of purified recombinant StWRKY1 protein on 12% SDS-PAGE. (B) EMSA showing DNA–protein interaction of the *THT* promoter with StWRKY1.

Figure S7. (A) Schematic representation of TRV-VIGS vectors. (B) Silencing of the *PDS* gene.

Table S1. Primer sequences used for quantifying RR gene expression in potato genotypes.

Table S2. RR metabolites detected in the resistant potato genotype following *P. infestans* or mock-solution inoculation.

Table S3. Promoter analysis of *RR* genes in the resistant potato genotype.

Table S4. Promoter analysis of StWRKY1 gene in resistant and susceptible potato genotypes.

Table S5. The differentially expressed HSPs detected in potato following *P. infestans* inoculation using RNA sequencing analysis.

Supplementary Data

## References

[CIT0001] Amin J Ananthan J Voellmy R 1988 Key features of heat shock regulatory elements. Molecular and Cellular Biology 8, 3761–3769. 3146692 10.1128/mcb.8.9.3761-3769.1988 PMC365434

[CIT0002] Alemanno L Ramos T Gargadenec A Andary C Ferriere N 2003 Localization and identification of phenolic compounds in *Theobroma cacao* L. somatic embryogenesis. Annals of Botany 92, 613–623. 12933367 10.1093/aob/mcg177 PMC4243679

[CIT0003] Alves MS Dadalto SP Gonçalves AB de Souza GB Barros VA Fietto LG 2014 Transcription factor functional protein-protein interactions in plant defense responses. Proteomes 2, 85–106. 10.3390/proteomes2010085 PMC5302731 28250372

[CIT0004] Asai S Ohta K Yoshioka H 2008 MAPK signaling regulates nitric oxide and NADPH oxidase-dependent oxidative bursts in *Nicotiana benthamiana* . The Plant Cell Online 20, 1390–1406. 10.1105/tpc.107.055855 PMC2438462 18515503

[CIT0005] Banzet N Richaud C Deveaux Y Kazmaier M Gagnon J Triantaphylidès C 1998 Accumulation of small heat shock proteins, including mitochondrial HSP22, induced by oxidative stress and adaptive response in tomato cells. The Plant Journal 13, 519–527 9680997 10.1046/j.1365-313x.1998.00056.x

[CIT0006] Chantreau M Chabbert B Billiard S Hawkins S Neutelings G 2015 Functional analyses of cellulose synthase genes in flax (*Linum usitatissimum*) by virus‐induced gene silencing. Plant Biotechnology Journal , 1–13. 25688574 10.1111/pbi.12350

[CIT0007] Chisholm ST Coaker G Day B Staskawicz BJ 2006 Host-microbe interactions: shaping the evolution of the plant immune response. Cell 124, 803–814. 16497589 10.1016/j.cell.2006.02.008

[CIT0008] Chujo T Miyamoto K Shimogawa T Shimizu T Otake Y Yokotani N Nishizawa Y Shibuya N Nojiri H Yamane H 2013 OsWRKY28, a PAMP-responsive transrepressor, negatively regulates innate immune responses in rice against rice blast fungus. Plant Molecular Biology 82, 23–37. 23462973 10.1007/s11103-013-0032-5

[CIT0009] Cosgrove DJ 2005 Growth of the plant cell wall. Nature Reviews Molecular Cell Biology 6, 850–861. 16261190 10.1038/nrm1746

[CIT0010] Czernic P Visser B Sun W Savouré A Deslandes L Marco Y Van Montagu M Verbruggen N 1999 Characterization of an *Arabidopsis thaliana* receptor‐like protein kinase gene activated by oxidative stress and pathogen attack. The Plant Journal 18, 321–327. 10377997 10.1046/j.1365-313x.1999.00447.x

[CIT0011] Dang F Wang Y She J Lei Y Liu Z Eulgem T Lai Y Lin J Yu L Lei D 2014 Overexpression of CaWRKY27, a subgroup IIe WRKY transcription factor of *Capsicum annuum*, positively regulates tobacco resistance to *Ralstonia solanacearum* infection. Physiologia Plantarum 150, 397–411. 24032447 10.1111/ppl.12093

[CIT0012] Dangl JL Horvath DM Staskawicz BJ 2013 Pivoting the plant immune system from dissection to deployment. Science 341, 746–751. 23950531 10.1126/science.1236011 PMC3869199

[CIT0013] De Vos RC Moco S Lommen A Keurentjes JJ Bino RJ Hall RD 2007 Untargeted large-scale plant metabolomics using liquid chromatography coupled to mass spectrometry. Nature Protocols 2, 778–791. 17446877 10.1038/nprot.2007.95

[CIT0014] Dellagi A Heilbronn J Avrova AO Montesano M Palva ET Stewart HE Toth IK Cooke DE Lyon GD Birch PR 2000 A potato gene encoding a WRKY-like transcription factor is induced in interactions with *Erwinia carotovora* subsp. *atroseptica* and *Phytophthora infestans* and is coregulated with class I endochitinase expression. Molecular Plant–Microbe Interactions 13, 1092–1101. 11043470 10.1094/MPMI.2000.13.10.1092

[CIT0015] Franke RB Dombrink I Schreiber L 2012 Suberin goes genomics: use of a short living plant to investigate a long lasting polymer. Frontiers in Plant Science 3, 4. 22639633 10.3389/fpls.2012.00004 PMC3355613

[CIT0016] Fry W 2008 *Phytophthora infestans*: the plant (and R gene) destroyer. Molecular Plant Pathology 9, 385–402. 18705878 10.1111/j.1364-3703.2007.00465.x PMC6640234

[CIT0017] Gunnaiah R Kushalappa AC Duggavathi R Fox S Somers DJ 2012 Integrated metabolo-proteomic approach to decipher the mechanisms by which wheat QTL (Fhb1) contributes to resistance against *Fusarium graminearum* . PloS One 7, e40695. 22866179 10.1371/journal.pone.0040695 PMC3398977

[CIT0018] Haslbeck M Vierling E 2015 A first line of stress defence: small heat shock proteins and their function in protein homeostasis. Journal of Molecular Biology 427, 1537–1548. 25681016 10.1016/j.jmb.2015.02.002 PMC4360138

[CIT0019] Hoffmann L Besseau S Geoffroy P Ritzenthaler C Meyer D Lapierre C Pollet B Legrand M 2004 Silencing of hydroxycinnamoyl-coenzyme A shikimate/quinate hydroxycinnamoyltransferase affects phenylpropanoid biosynthesis. The Plant Cell Online 16, 1446–1465. 10.1105/tpc.020297 PMC490038 15161961

[CIT0020] Jones JD Dangl JL 2006 The plant immune system. Nature 444, 323–329. 17108957 10.1038/nature05286

[CIT0021] Katou S Yamamoto A Yoshioka H Kawakita K Doke N 2003 Functional analysis of potato mitogen-activated protein kinase kinase, StMEK1. Journal of General Plant Pathology 69, 161–168.

[CIT0022] Kim CY Zhang S 2004 Activation of a mitogen‐activated protein kinase cascade induces WRKY family of transcription factors and defense genes in tobacco. The Plant Journal 38, 142–151. 15053767 10.1111/j.1365-313X.2004.02033.x

[CIT0023] Kumar A Dutt S Bagler G Ahuja PS Kumar S 2012 Engineering a thermo-stable superoxide dismutase functional at sub-zero to >50°C, which also tolerates autoclaving. Scientific Reports 2, 387. 22548128 10.1038/srep00387 PMC3339387

[CIT0024] Kumar A Kaachra A Bhardwaj S Kumar S 2014 Copper, zinc superoxide dismutase of *Curcuma aromatica* is a kinetically stable protein. Process Biochemistry 49, 1288–1296.

[CIT0025] Kushalappa AC Gunnaiah R 2013 Metabolo-proteomics to discover plant biotic stress resistance genes. Trends in Plant Science 18, 522–531. 23790252 10.1016/j.tplants.2013.05.002

[CIT0026] Laxalt AM Munnik T 2002 Phospholipid signalling in plant defence. Current Opinion in Plant Biology 5, 332–338. 12179967 10.1016/s1369-5266(02)00268-6

[CIT0027] Liu Y Schiff M Marathe R Dinesh‐Kumar S 2002 Tobacco Rar1, EDS1 and NPR1/NIM1 like genes are required for N‐mediated resistance to tobacco mosaic virus. The Plant Journal 30, 415–429. 12028572 10.1046/j.1365-313x.2002.01297.x

[CIT0028] Liu X Khajuria C Li J Trick HN Huang L Gill BS Reeck GR Antony G White FF Chen MS 2013 Wheat Mds-1 encodes a heat-shock protein and governs susceptibility towards the Hessian fly gall midge. Nature Communications 4, 2070. 10.1038/ncomms3070 23792912

[CIT0029] Livak KJ Schmittgen TD 2001 Analysis of relative gene expression data using real-time quantitative PCR and the 2^−ΔΔCT^ method. Methods 25, 402–408. 11846609 10.1006/meth.2001.1262

[CIT0030] Machinandiarena MF Lobato MC Feldman ML Daleo GR Andreu AB 2012 Potassium phosphite primes defense responses in potato against *Phytophthora infestans* . Journal of Plant Physiology 169, 1417–1424. 22727804 10.1016/j.jplph.2012.05.005

[CIT0031] Maimbo M Ohnishi K Hikichi Y Yoshioka H Kiba A 2007 Induction of a small heat shock protein and its functional roles in *Nicotiana* plants in the defense response against *Ralstonia solanacearum* . Plant Physiology 145, 1588–1599. 17965181 10.1104/pp.107.105353 PMC2151688

[CIT0032] Marchive C Léon C Kappel C Coutos-Thévenot P Corio-Costet M-F Delrot S Lauvergeat V 2013 Over-Expression of VvWRKY1 in grapevines induces expression of jasmonic acid pathway-related genes and confers higher tolerance to the downy mildew. PloS One 8, e54185. 23342101 10.1371/journal.pone.0054185 PMC3544825

[CIT0033] Nicot N Hausman J-F Hoffmann L Evers D 2005 Housekeeping gene selection for real-time RT-PCR normalization in potato during biotic and abiotic stress. Journal of Experimental Botany 56, 2907–2914. 16188960 10.1093/jxb/eri285

[CIT0034] Petitot A-S Barsalobres-Cavallari C Ramiro D Freire EA Etienne H Fernandez D 2013 Promoter analysis of the WRKY transcription factors CaWRKY1a and CaWRKY1b homoeologous genes in coffee (*Coffea arabica*). Plant Cell Reports 32, 1263–1276. 23568411 10.1007/s00299-013-1440-3

[CIT0035] Pluskal T Castillo S Villar-Briones A Orešič M 2010 MZmine 2: modular framework for processing, visualizing, and analyzing mass spectrometry-based molecular profile data. BMC Bioinformatics 11, 395. 20650010 10.1186/1471-2105-11-395 PMC2918584

[CIT0036] **Potato Genome Sequencing Consortium**. 2011 Genome sequence and analysis of the tuber crop potato. Nature 475, 189–195. 21743474 10.1038/nature10158

[CIT0037] Pushpa D Yogendra KN Gunnaiah R Kushalappa AC Murphy A 2014 Identification of late blight resistance-related metabolites and genes in potato through nontargeted metabolomics. Plant Molecular Biology Reporter 32, 584–595.

[CIT0038] Rejeb IB Pastor V Mauch-Mani B 2014 Plant responses to simultaneous biotic and abiotic stress: molecular mechanisms. Plants 3, 458–475. 10.3390/plants3040458 PMC4844285 27135514

[CIT0039] Robert-Seilaniantz A Grant M Jones JD 2011 Hormone crosstalk in plant disease and defense: more than just jasmonate-salicylate antagonism. Annual Review of Phytopathology 49, 317–343. 10.1146/annurev-phyto-073009-114447 21663438

[CIT0040] Rushton PJ Somssich IE Ringler P Shen QJ 2010 WRKY transcription factors. Trends in Plant Science 15, 247–258. 20304701 10.1016/j.tplants.2010.02.006

[CIT0041] Shan Q Wang Y Li J Gao C 2014 Genome editing in rice and wheat using the CRISPR/Cas system. Nature Protocols 9, 2395–2410. 25232936 10.1038/nprot.2014.157

[CIT0042] Storozhenko S De Pauw P Van Montagu M Inzé D Kushnir S 1998 The heat-shock element is a functional component of the *Arabidopsis* APX1 gene promoter. Plant Physiology 118, 1005–1014. 9808745 10.1104/pp.118.3.1005 PMC34773

[CIT0043] Van Ooijen G Lukasik E Van Den Burg HA Vossen JH Cornelissen BJ Takken FL 2010 The small heat shock protein 20 RSI2 interacts with and is required for stability and function of tomato resistance protein I‐2. The Plant Journal 63, 563–572. 20497382 10.1111/j.1365-313X.2010.04260.x PMC2988412

[CIT0044] Walter M Chaban C Schütze K Batistic O Weckermann K Näke C Blazevic D Grefen C Schumacher K Oecking C 2004 Visualization of protein interactions in living plant cells using bimolecular fluorescence complementation. The Plant Journal 40, 428–438. 15469500 10.1111/j.1365-313X.2004.02219.x

[CIT0045] Wang X Fan C Zhang X Zhu J Fu Y-F 2013 BioVector, a flexible system for gene specific-expression in plants. BMC Plant Biology 13, 198. 24304941 10.1186/1471-2229-13-198 PMC4235170

[CIT0046] Wang Y Dang F Liu Z Wang X Eulgem T Lai Y Yu L She J Shi Y Lin J 2013 CaWRKY58, encoding a group I WRKY transcription factor of *Capsicum annuum*, negatively regulates resistance to *Ralstonia solanacearum* infection. Molecular Plant Pathology 14, 131–144. 23057972 10.1111/j.1364-3703.2012.00836.x PMC6638745

[CIT0047] War AR Paulraj MG Ahmad T Buhroo AA Hussain B Ignacimuthu S Sharma HC 2012 Mechanisms of plant defense against insect herbivores. Plant Signaling & Behavior 7, 1306–1320. 22895106 10.4161/psb.21663 PMC3493419

[CIT0048] Wu C Avila CA Goggin FL 2015 The ethylene response factor Pti5 contributes to potato aphid resistance in tomato independent of ethylene signalling. Journal of Experimental Botany 66, 559–570. 25504643 10.1093/jxb/eru472 PMC4286409

[CIT0049] Ye S Jiang Y Duan Y Karim A Fan D Yang L Zhao X Yin J Luo K 2014 Constitutive expression of the poplar WRKY transcription factor PtoWRKY60 enhances resistance to *Dothiorella gregaria* Sacc. in transgenic plants. Tree Physiology 34, 1118–1129. 25281841 10.1093/treephys/tpu079

[CIT0050] Yogendra KN Kushalappa AC Sarmiento F Rodriguez E Mosquera T 2015 Metabolomics deciphers quantitative resistance mechanisms in diploid potato clones against late blight. Functional Plant Biology 42, 284–298. 10.1071/FP14177 32480674

[CIT0051] Yogendra KN Pushpa D Mosa KA Kushalappa AC Murphy A Mosquera T 2014 Quantitative resistance in potato leaves to late blight associated with induced hydroxycinnamic acid amides. Functional & Integrative Genomics 14, 285–298. 24408130 10.1007/s10142-013-0358-8

[CIT0052] Yokotani N Sato Y Tanabe S Chujo T Shimizu T Okada K Yamane H Shimono M Sugano S Takatsuji H 2013 WRKY76 is a rice transcriptional repressor playing opposite roles in blast disease resistance and cold stress tolerance. Journal of Experimental Botany 64, 5085–5097. 24043853 10.1093/jxb/ert298 PMC3830488

