# Supplementary material for: Transcription factor StWRKY1 regulates phenylpropanoid metabolites conferring late blight resistance in potato
Source: J Exp Bot. 2015 Sep 28;66(22):7377–89. doi: 10.1093/jxb/erv434 (PMC4765800; doi:10.1093/jxb/erv434)
Supplement: Supplementary Data [file supp_66_22_7377__index.html]

Transcription factor StWRKY1 regulates phenylpropanoid metabolites conferring late blight resistance in potato — Transcription factor StWRKY1 regulates phenylpropanoid metabolites conferring late blight resistance in potato — Supplementary Data 

# Transcription factor *StWRKY1* regulates phenylpropanoid metabolites conferring late blight resistance in potato

## Supplementary Data

Data files

- Supplementary Data - Supplementary Data
