# Supplementary material for: Transcription factor StWRKY1 regulates phenylpropanoid metabolites conferring late blight resistance in potato
Source: J Exp Bot. 2015 Sep 28;66(22):7377–89. doi: 10.1093/jxb/erv434 (PMC4765800; doi:10.1093/jxb/erv434)
Supplement: Supplementary Data [file supp_erv434_jexbot155895_file001.pdf]

The transcription factor *StWRKY1* regulates secondary cell wall thickening metabolites in potato resistant to late blight

Kalenahalli N. Yogendra, Arun Kumar, Kobir Sarkar, Yunliang Li, Pushpa Doddaraju, Kareem A. Mosa, Raj Duggavathi and Ajjamada C. Kushalappa

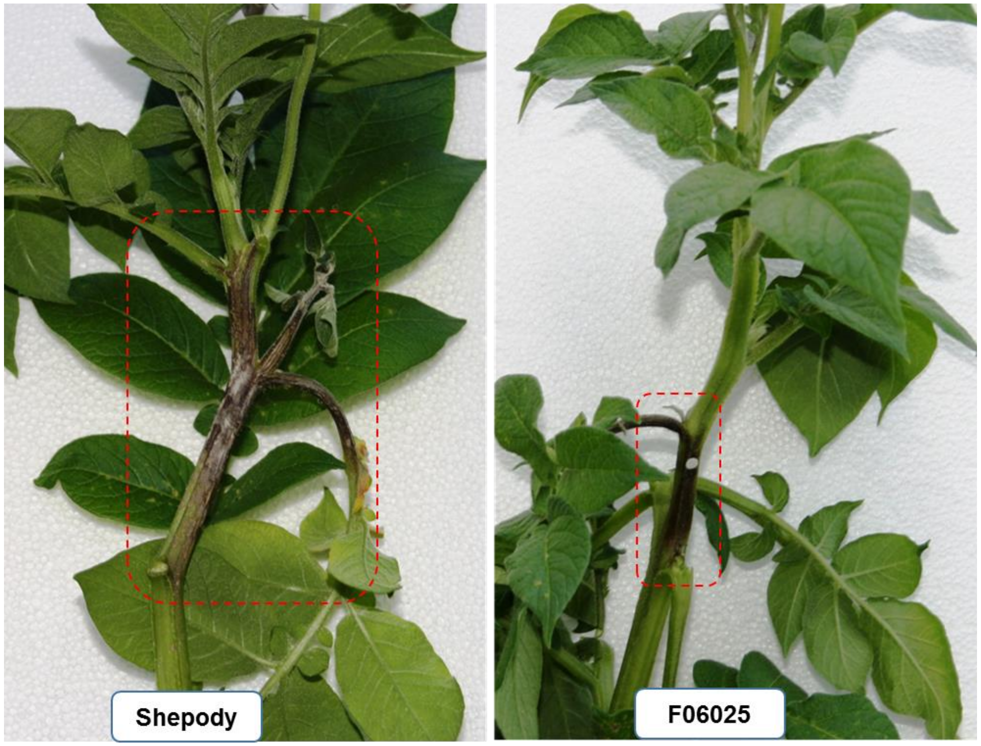

Fig. S1. *Phytophthora infestans* infected potato stems with susceptible (Shepody) and resistant (F06025) genotypes, at 9 dpi.

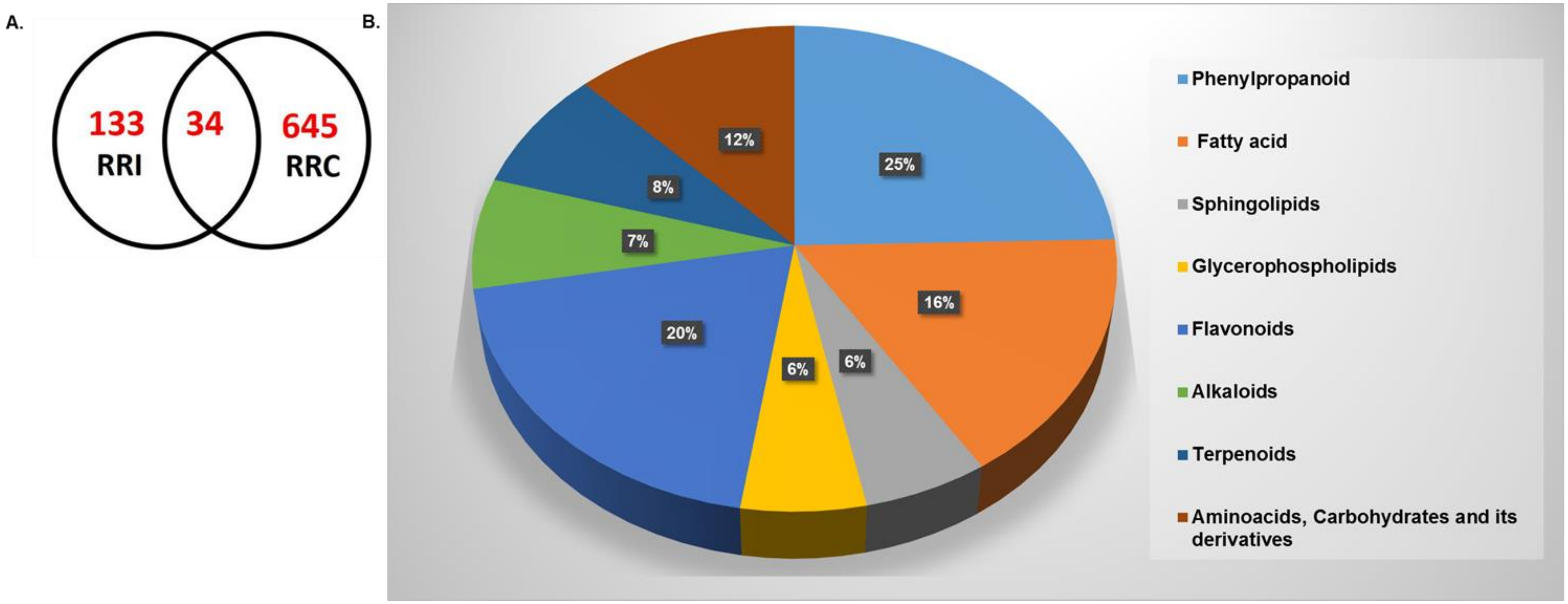

Fig. S2. (A) The number of resistance related metabolites identified in stem of resistant potato genotype inoculated with water or *Phytophthora infestans*. Acronyms: RRC, resistance related constitutive; RRI, resistance related induced metabolites; (B) Resistance related (RR) metabolites identified in the resistant potato genotype, inoculated with mock-solution or *P. infestans* and their classification according to chemical groups.

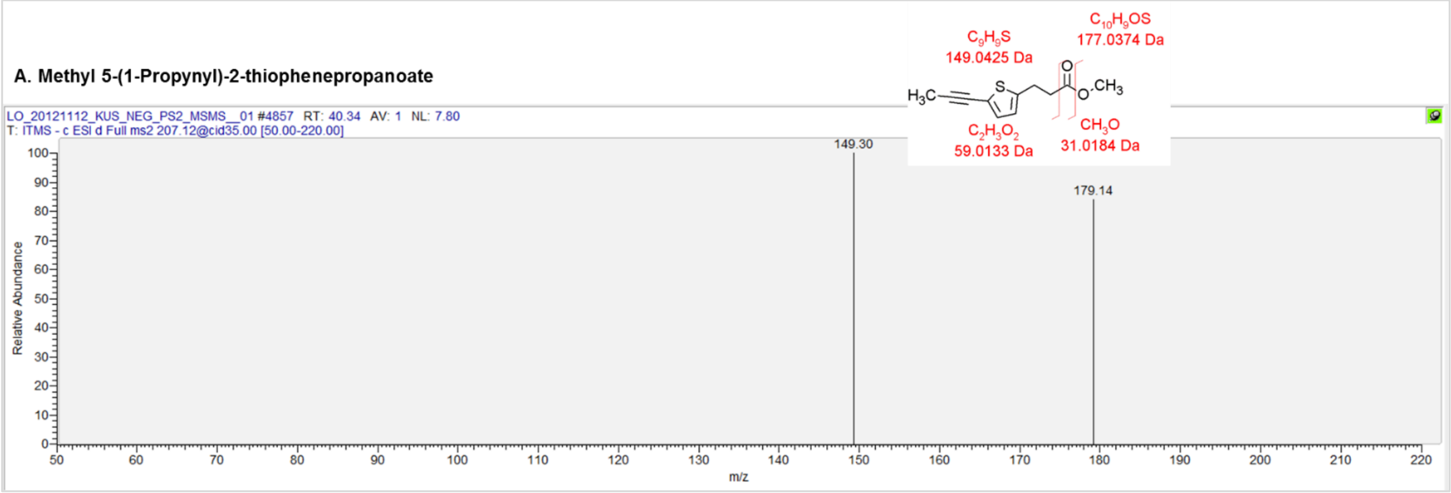
A. Methyl 5-(1-Propynyl)-2-thiophenepropanoate
LO_20121112_KUS_NEG_PS2_MSMS__01 #4857 RT: 40.34 AV: 1 NL: 7.80
T: ITMS - c ESI d Full ms2 207.12@cid35.00 [50.00-220.00]
C_9H_9S
149.0425 Da
$C_{10}H_9OS$
177.0374 Da
$C_2H_3O_2$
59.0133 Da
CH_3O
31.0184 Da
149.30
179.14
Relative Abundance
m/z

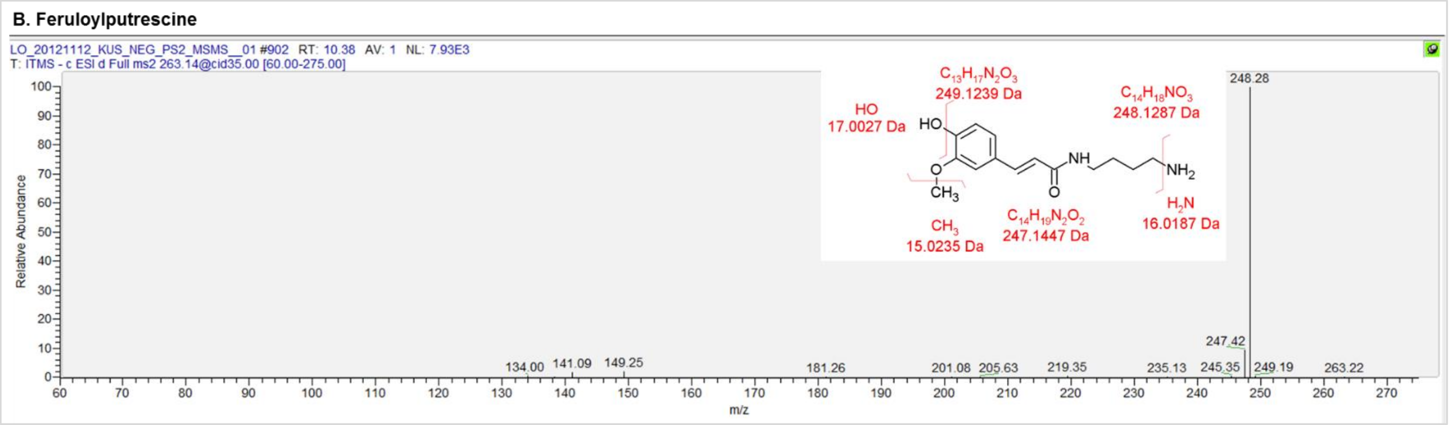
B. Feruloylputrescine
LO_20121112_KUS_NEG_PS2_MSMS__01 #902 RT: 10.38 AV: 1 NL: 7.93E3
T: ITMS - c ESI d Full ms2 263.14@cid35.00 [60.00-275.00]
$C_{13}H_{17}N_2O_3$
249.1239 Da
HO
17.0027 Da
$C_{14}H_{18}NO_3$
248.1287 Da
CH_3
15.0235 Da
$C_{14}H_{19}N_2O_2$
247.1447 Da
H_2N
16.0187 Da
248.28
247.42
134.00 141.09 149.25 181.26 201.08 205.63 219.35 235.13 245.35 249.19 263.22
Relative Abundance
m/z

C. Caffeic acid 3-glucoside

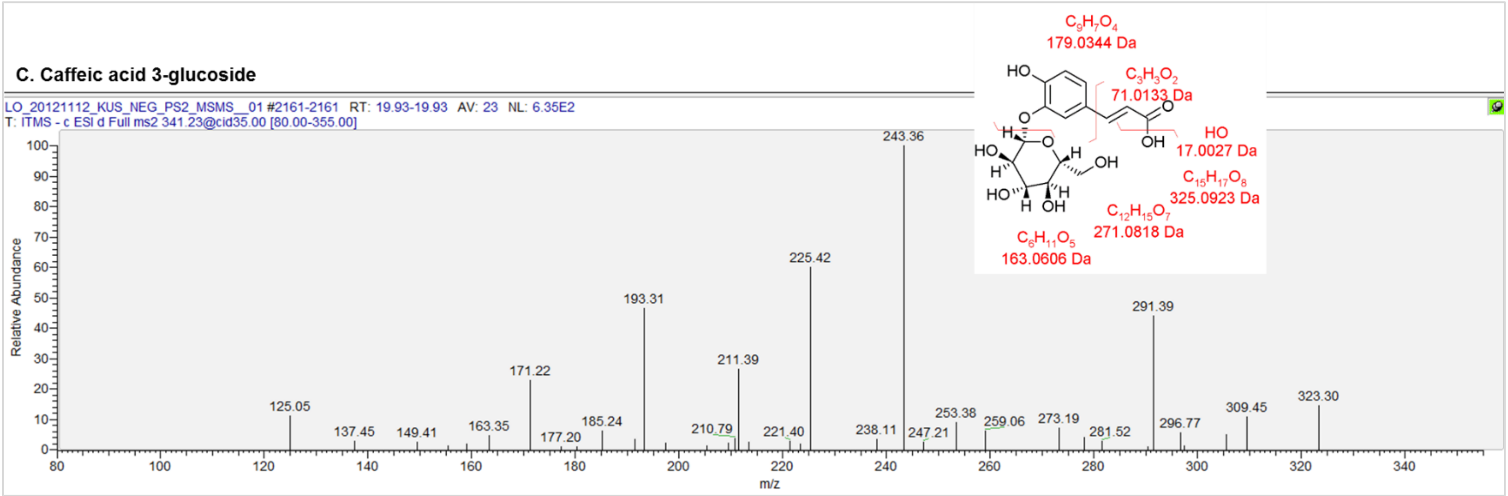

D. 10-Acetoxyligustroside

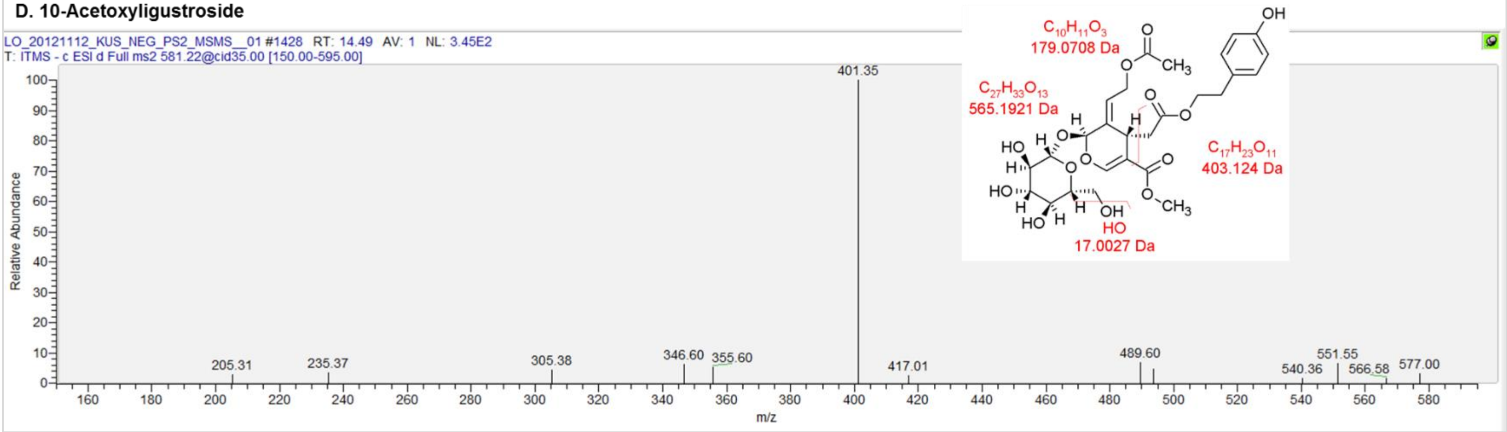

E. 1,2-di-O-sinapoyl-β-D-glucose

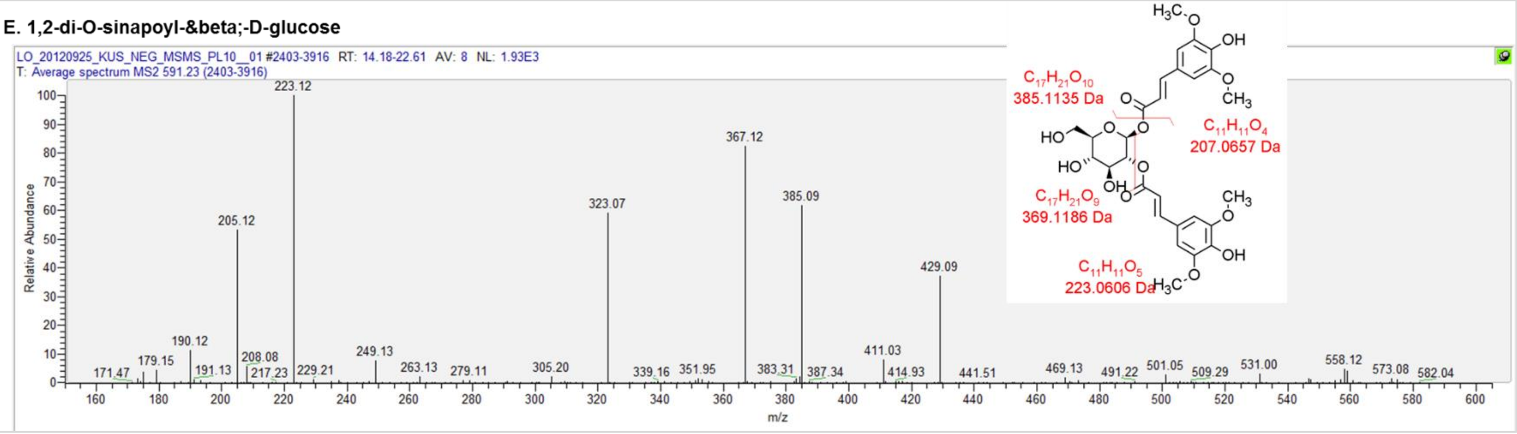

F. Cer(d14:2(4E,6E)/16:0)

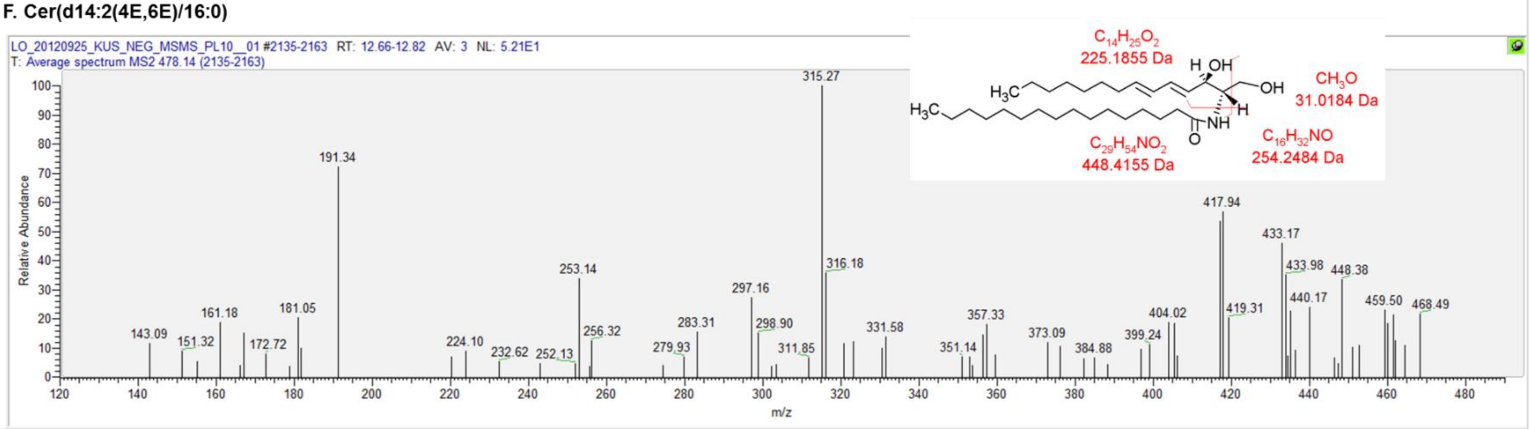

G. Cer(d18:1/16:0)

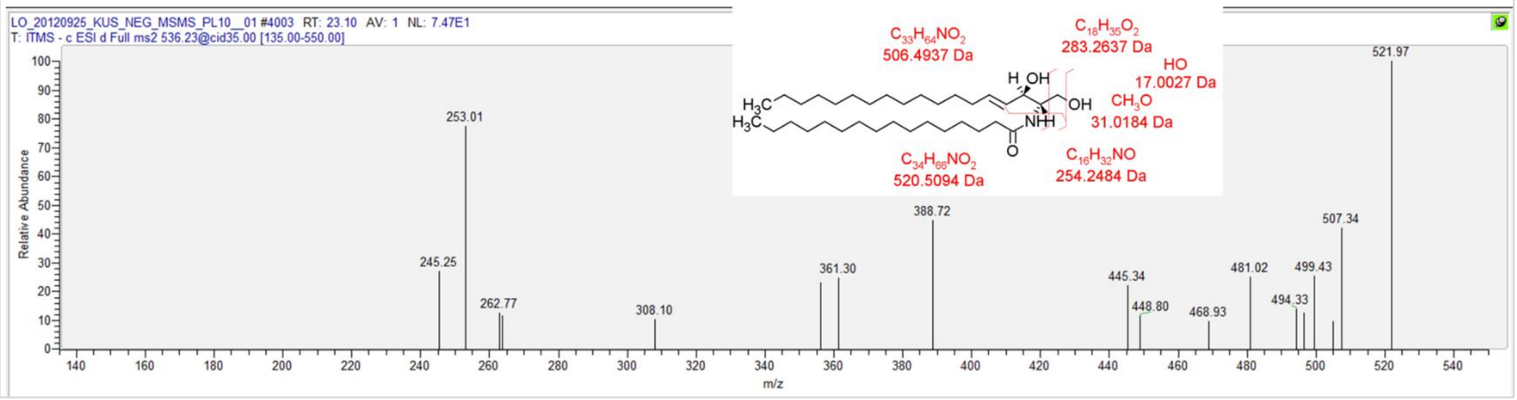

H. Cer(d18:0/20:0)

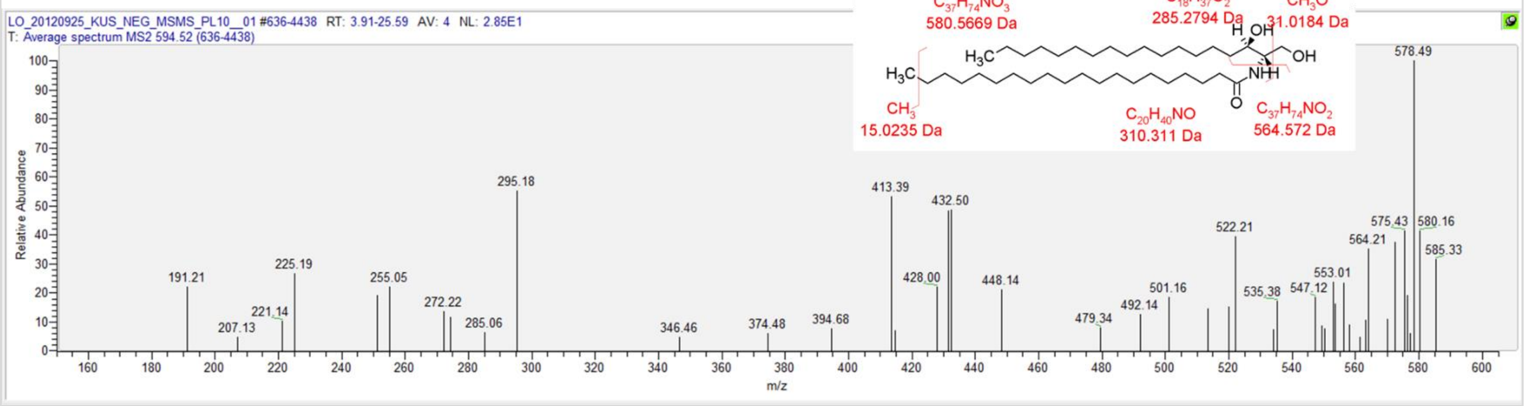

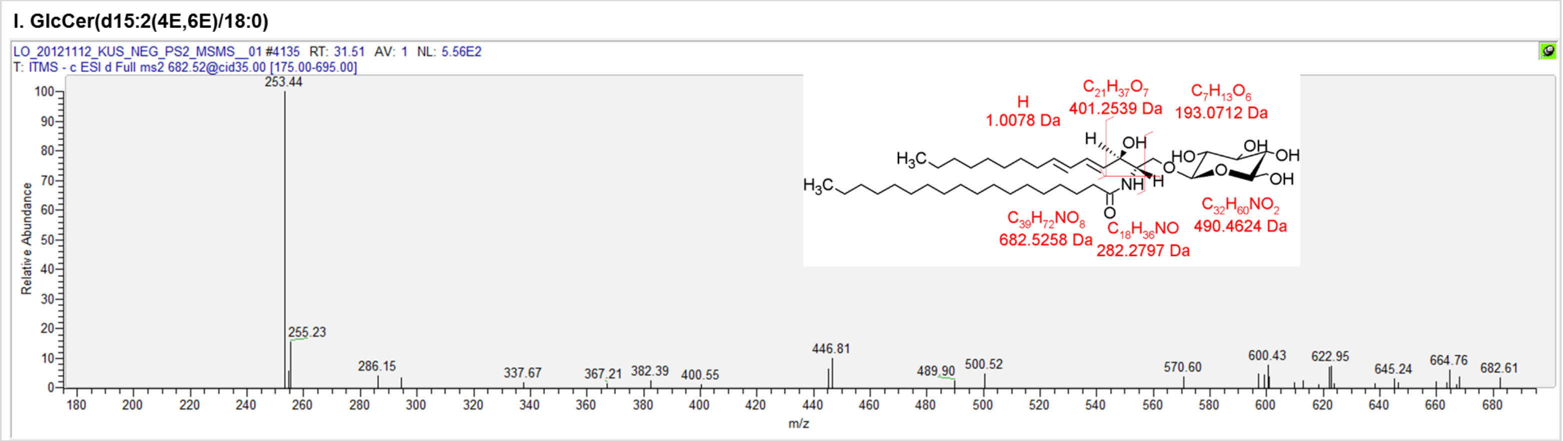

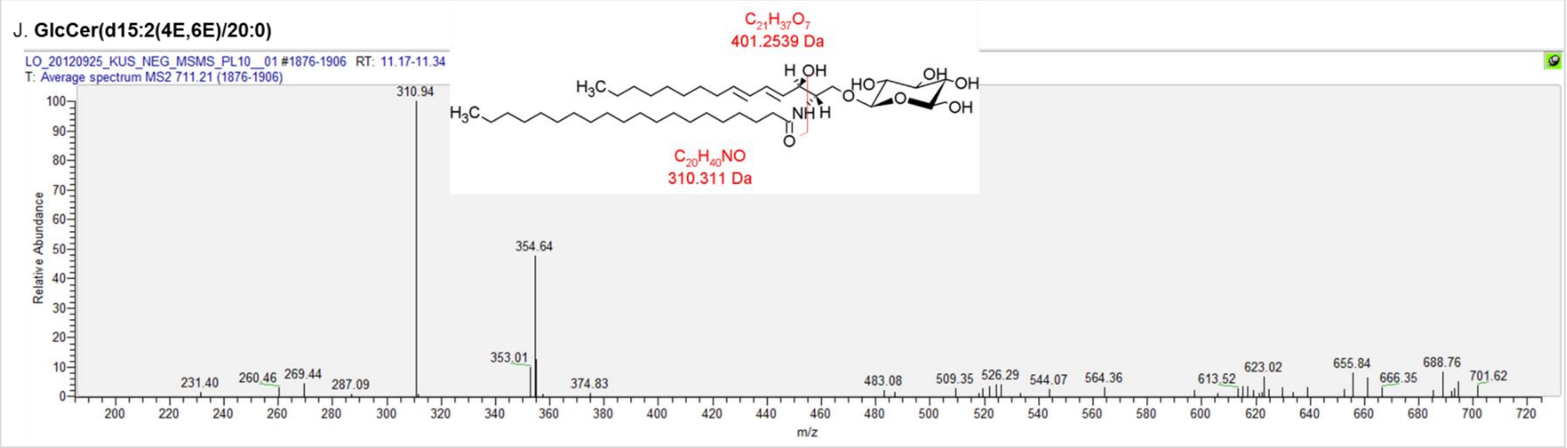

Fig. S3. *In silico* fragmentation of resistance related metabolites

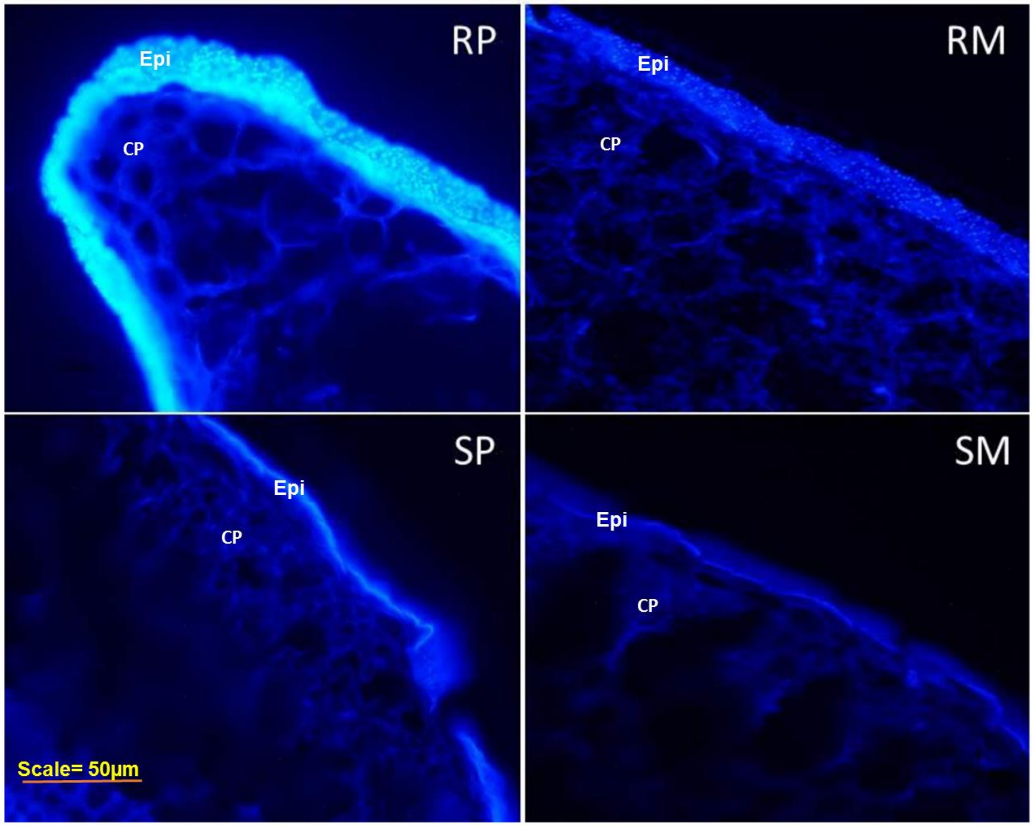

Fig. S4. Laser scanning confocal micrographs of stem sections, exhibiting secondary cell wall thickening, due to hydroxycinnamic acid amides (HCAAs- blue fluorescence). RP is resistant genotype with *P. infestans* inoculation, RM is resistant genotype with mock inoculation, SP is susceptible genotype with *P. infestans* inoculation, SM is susceptible genotype with mock inoculation. Epi is epidermis, CP is cortical parenchyma.

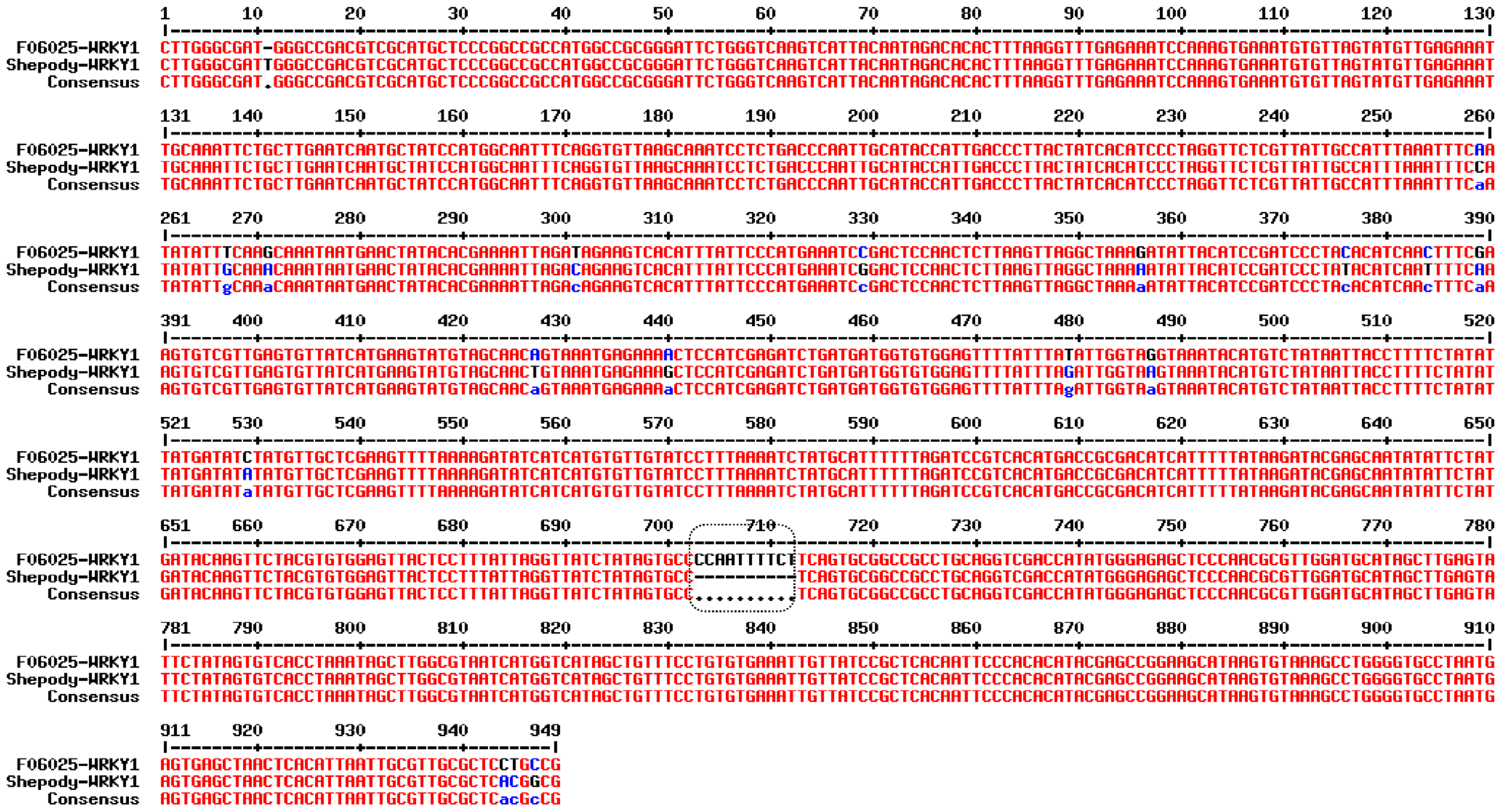

Fig. S5. Comparison of sequence variation between resistant (F06025) and susceptible (Shepody) StWRKY1 promoter region.

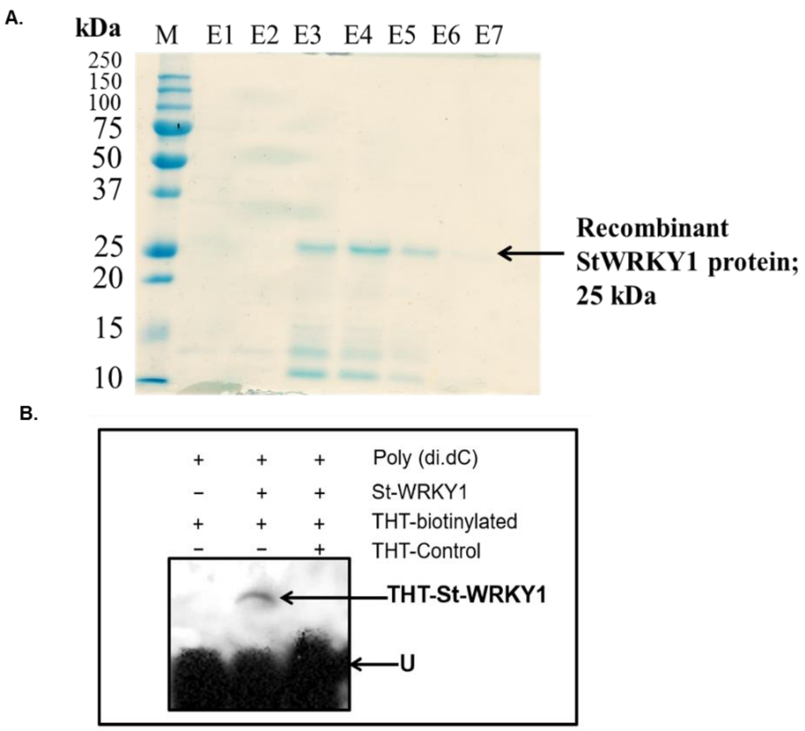

Fig. S6. (A) Analysis of purified recombinant StWRKY1 protein on 12% Sodium dodecyl sulphate-Polyacrylamide gel electrophoresis. StWRKY1 was cloned in pTrc HisB vector (Invitrogen, USA), overexpressed in *Escherichia coli* BL21 cells and was purified by affinity chromatography using nickel NTA as matrix. Different protein fraction were loaded and electrophoresed in 12% SDS-polyacrylamide gel and were stained with Sigma EZBlue™ Gel Staining Reagent (Sigma, USA). M, Precision plus Protein™ Prestained Marker (Biorad, Canada); E1 to E7 represent various eluted protein fractions. (B) Electrophoretic mobility gel shift assay (EMSA) showing DNA-protein interaction of THT promoter with StWRKY1. The position of the specifically retarded DNA/protein complex (THT-WRKY1) and that of the free probe (U) is also shown on the right side of the panel with an arrow mark.

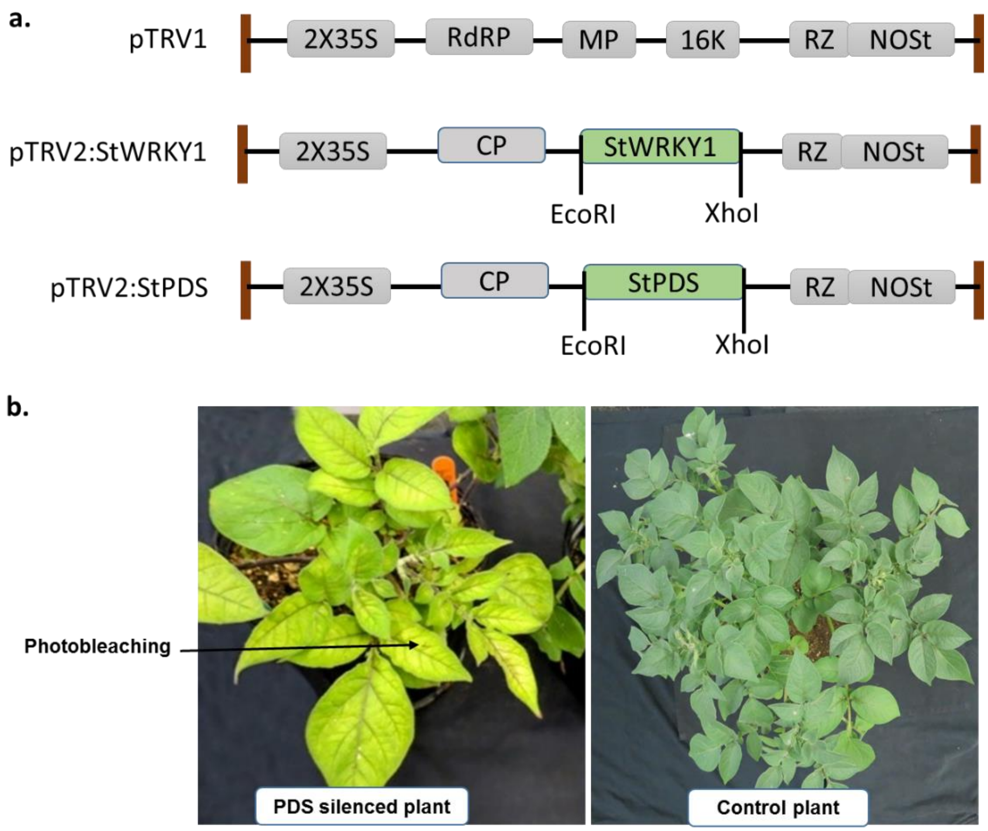

Fig. S7. (A) Schematic representation of tobacco rattle virus (TRV) based virus induced gene silencing (VIGS) vectors. TRV cDNA clones were placed in between the duplicated cauliflower mosaic virus (CaMV) 35S promoter (2 X 35S) and the nopaline synthase terminator (NOS) in a T-DNA vector. LB and RB refer to left and right borders of T-DNA. Rz, self-cleaving ribozyme. (b) Silencing of the phytoene desaturase (PDS) gene. Infection of recombinant TRV carrying the PDS sequence silences endogenous PDS in potato plants and causes inhibition of carotenoid biosynthesis resulting in the photobleaching phenotype.

Table S1. Primer sequences used for quantifying resistance related gene expression in potato genotypes

Genbank name	Gene name	Forward primer (5'-3')	Reverse primer (5'-3')
**A. Biomass quantification**			
GQ371195.1	O-8	GAAAGGCATAGAAGGTAGA	TAACCGACCAAGTAGTAAA
AB061263.1	StEf1-α	ATTGGAAACGGATATGCTCCA	TCCTTACCTGAACGCCTGTCA
Z33382.1	b-tubulin	ATGTTCAGGCGCAAGGCTT	TCTGCAACCGGGTCATTCAT
**B. qRT-PCR primers**			
AF150686.1	St4CL	ATCCTGAGGCCACAGCTAGA	TTACGGGACAACAGCAGCAT
JX896425.1	StTHT	TGTTGTTGTCAGCTGTGGCT	TTTGCAAAGGCTTGAAGGGC
PGSC0003DMT400038501[a]	SpTyDC	AGTGGTGGTGGAGGTGTACT	AGGTCCCAGATAGGGTCGTT
AJ278507.1	StWRKY1	GAGGAAATATGGACAAAAGGCTG	TTTGTCAATGGGATGTGAATGC
AB061263.1	StEf1-α	ATTGGAAACGGATATGCTCCA	TCCTTACCTGAACGCCTGTCA
Z33382.1	b-tubulin	ATGTTCAGGCGCAAGGCTT	TCTGCAACCGGGTCATTCAT
**C. Primers for sequencing**			
AJ278507.1	StWRKY1	TCTAAAATGGAGAATTATGCAACAA	TTAAAAGGAAGTATAGATTTGCATCTGA
PGSC0003DMT400069966[a]	StWRKY1 promoter	CTGGGTCAAGTCATTACAATAGA	TTTCTTCATGGGGCACTATAGA
**D. Primers for BiFC assay**			
AJ278507.1	StWRKY	GTCGACATGGAGAATTATGCAACAAT	GAATTCAAAGGAAGTATAGATTTGCA
AB091780.1	StMEK1		
**E. Primers for LUC assay**			
AF150686.1	St4CL	CTCGAGGAGTGTCAATTTTGACCAATTTTT	GGATCCATCTGGGCATCTTGAGAGCTAA
JX896425.1	StTHT	CTCGAGTCATCGCCCACAAGTTATGA	GGATCCGGGAAAGTATTTAGTTAAATTTGGAGA
PGSC0003DMT400038501[a]	SpTyDC	CTCGAG CCCACCCCTATCGCCTAT	GGATCCTTTCAATTTGTGTGTATGTTGTCTT

[a] Solanaceae Genomics Resource, Michigan State University, USA.

Table S2. Resistance related detected in resistant potato genotype following *P. infestans* or mock-solution inoculation

Observed mass (Da)	Exact mass (Da)	RT (Min)	AME (ppm)	Metabolite names	Observed fragmentation	Database fragmentation	Database ID
**A. Phenylpropanoids**							
148.0530	148.0524	2.83	1.60	trans-Cinnamic acid	**148.20,**104.05,**103.30**, 91.48	**147.0, 103.05**, 101.04, 77.04	C00423
162.0320	162.0316	2.50	2.14	4-hydroxycoumarin	**161.02,** 131.06, **116.54**	**161.02, 117.03**, 41.00	CPD-12111
165.0793	165.0790	1.62	2.15	L-Phenylalanine	**163.90**,**149.05**,**147.12**, **120.22**, **72.05**	**163.9**, **148.8, 147.2**, 146.5, **120.1**, **71.9**	PHE
166.0633	166.0630	1.898	9.85	Dihydro-3-coumaric acid	**150.14, 149.01,** 123.14, 95.14	**149.00**, 121.06, 119.05, 106.04	CPD-12218
174.0533	174.0528	18.61	2.90	Shikimate	175.32,**157.11**,132.94, 131.05	**157.05**,17.00	C00493 ***In silico***
181.0742	181.0739	0.72	1.88	α-amino oxy-β-phenyl propionate	**163.19, 136.16, 119.16, 93.09**	**162.05, 135.04, 120.04, 119.04, 93.03**	CPD-7021
192.0273	192.0270	0.44	1.48	Citrate	**155.15, 148.12, 129.20, 111.00**	**154.60, 148.20, 129.20, 111.10**	CIT
192.0637	192.0634	5.83	1.50	Quinate	**191.07,173.07,171.08,126.98, 111.03,92.99**	**191.3, 173.4, 170.8, 127.0, 111.4, 93.0**	C00296
194.0585	194.0579	4.40	2.89	Ferulic acid	193.22,**178.34**,175.10, 173.13,	**178.02**,134.03,133.02	METLIN
208.0552	208.0558	23.47	2.71	Methyl 5-(1-Propynyl)-2-thiophenepropanoate	179.14, **149.30**	177.03, **149.04**	90185-Metlin ***In silico***
264.1472	264.1474	10.54	0.73	Feruloylputrescine	**249.19, 248.28, 247.42**	**249.12, 248.12, 247.14**	***In silico***
300.0844	300.0845	1.21	0.43	Salicylate β-D-glucose ester	217.05, 201.30, 161.33, **137.11**, 93.08	179.26, **137.15**	CPD-12629

306.1690	306.1692	12.35	0.64	Feruloylagmatine	288.23, **263.25**, 248.28, **149.12**, 134.15	289.14, **263.13**, 177.05, **149.06**	CPD-12236 (Gunnaiah *et al.*, 2012)
313.1317	313.1314	17.25	0.86	N-Feruloyltyramine	**178.13,** 177.12, 166.15, 148.24,	**178.0**,122,138	C02717 (Yogendra *et al.*, 2015)
329.1271	329.1263	15.18	2.45	N-Feruloyloctopamine	328.37,**313.76,312.43**, 269.20,	**314.10, 312.12**	METLIN (Yogendra et al., 2015)
342.0946	342.0951	8.26	1.45	Caffeic acid 3-glucoside	323.30, 273.19, 177.20, **163.35**	325.09, 271.08, 179.03, **163.06**	C10431 *In silico*
354.0958	354.0951	6.76	1.86	Scopolin	330.99,192.05,**191.08**, 178.96,	339.07, **191.03**, 163.06	C01527 (Yogendra et al., 2015)
368.1115	368.1107	8.58	2.11	5-O-Feruloylquinic acid	351.90,**193.13**,191.05	353.08, 351.10,**193.05**, 175.06	C02572
386.1215	386.1213	3.23	0.50	1-O-Sinapoyl-beta-D-glucose	**247.23, 223.04, 205.19**	**247.06, 223.06, 205.04**	C01175
582.1941	582.1949	14.24	1.32	10-Acetoxyligustroside	**566.58, 401.35**	**565.19, 403.12, 179.07**	90532-Metlin *In silico*
592.1790	592.1792	13.90	0.32	1,2-di-O-sinapoyl-β-D-glucose	429.23, **385.18**, 367.15, 323.07, **223.16**, 205.15	**385.11**, 369.11, **223.06,** 207.06	12-BIS-O-SINAPOYL-BETA-D-GLUCOSIDE
**B. Sphingolipids**							
479.4357	479.4338	30.83	3.87	Cer(d14:2(4E,6E)/16:0)	**448.38, 253.14,** 252.13, **224.10**	**448.41, 254.24, 225.18**	LMSP02010051 *In silico*
537.5138	537.5121	23.48	3.10	Cer(d18:1/16:0)	**521.97, 507.34,** 388.72, **253.01**	**520.50, 506.49,** 283.26, **254.24**	LMSP02010004 *In silico*

595.5880	595.5903	36.05	3.86	Cer(d18:0/20:0)	**580.16,** 578.49, **564.21, 285.06**	**580.56, 564.57,** 310.31, **285.27**	LMSP02020009 *In silico*
683.5331	683.5336	30.81	0.79	GlcCer(d15:2(4E,6E)/18:0)	**682.61, 489.90, 400.55,** 286.15	**682.52, 490.46, 401.25, 282.27**	LMSP0501AA58 *In silico*
711.5643	711.5649	30.11	0.89	GlcCer(d15:2(4E,6E)/20:0)	354.64, 353.01, **310.94**	401.25, **310.31**	LMSP0501AA59
851.5776	851.5793	23.52	2.04	(3'-sulfo)Galbeta-Cer(d18:0/20:0(2OH))	704.41, 522.05, **325.41**	525.27, **326.30**	LMSP06020005 *In silico*
**C. Glycerophospholipids**							
537.3090	537.3067	23.46	4.37	1-(9Z-nonadecenoyl)-glycero-3-phosphoserine	**536.21, 520.84, 339.37, 197.29**	**536.29, 522.28, 339.28, 198.01**	LMGP03050019
537.3790	537.3794	23.48	0.82	1-hexadecyl-2-propionyl-sn-glycero-3-phosphocholine	455.12, **354.30, 182.98**	522.35, 464.35, **355.32, 182.05**	LMGP01020068
537.4151	537.4158	23.48	1.36	1-octadecyl-2-ethyl-sn-glycero-3-phosphocholine	**493.57,** 455.12	**492.38**	LMGP01040053

AME: Accurate Mass Error= ((Observed mass - expected mass) / expected mass) X 10^6, RT: Retention time

Database ID examples: Number-METLIN, LMP-LIPIDMAPS, KEGG-C05610, KNAPSACK- C00002775, HMDB-HMDB33026, and PMN-Plant Metabolic Network, *In silico*: In silico fragmentation

References

Gunnaiah, R., Kushalappa, A. C., Duggavathi, R., Fox, S. and Somers, D. J. (2012) Integrated metabolo-proteomic approach to decipher the mechanisms by which wheat QTL (Fhb1) contributes to resistance against Fusarium graminearum. *PloS one,* **7,** e40695.

Yogendra, K. N., Kushalappa, A. C., Sarmiento, F., Rodriguez, E. and Mosquera, T. (2015) Metabolomics deciphers quantitative resistance mechanisms in diploid potato clones against late blight. *Functional Plant Biology,* **42,** 284-298.

Table S3. Promoter analysis of resistance related gene in resistant potato genotype

4-CL promoter	THT promoter	TYDC promoter	HCT promoter
4cl-CMA2a: light responsive element	3-AF1 binding site: light responsive element	ABRE: cis-acting element involved in the abscisic acid responsiveness	3-AF3 binding site: light responsive element
4cl-CMA2b: light responsive element	ACE: cis-acting element involved in light responsiveness	ARE: cis-acting regulatory element essential for the anaerobic induction	AACA_motif: involved in endosperm-specific negative expression
AC-I: negative regulation of phloem expression	AE-box: part of a module for light response	Box I: light responsive element	AC-I: negative regulation of phloem expression
AC-II: enhanced xylem expression and repressed phloem	ARE: cis-acting regulatory element essential for the anaerobic induction	Box 4: light responsive element	ACE: cis-acting element involved in light responsiveness
**Box-W1: WRKY1 protein binding site**	**Box-W1: WRKY1 protein binding site**	**Box-W1: WRKY1 protein binding site**	ARE: cis-acting regulatory element essential for the anaerobic induction
CAAT-box: common cis-acting element in promoter and enhancer regions	Box I: light responsive element	CAAT-box: common cis-acting element in promoter and enhancer regions	Box I: light responsive element
GA-motif: part of a light responsive element	CAAT-box: common cis-acting element in promoter and enhancer regions	CGTCA-motif: cis-acting regulatory element involved in the MeJA-responsiveness	CAAT-box: common cis-acting element in promoter and enhancer regions
L-box: part of a light responsive element	CGTCA-motif: cis-acting regulatory element involved in the MeJA-responsiveness	ERE: ethylene-responsive element	ERE: ethylene-responsive element

OBP-1 site: cis-acting regulatory element	ELI-box3: elicitor-responsive element	G-Box: cis-acting regulatory element involved in light responsiveness	G-Box: cis-acting regulatory element involved in light responsiveness
TATA-box: core promoter element around -30 of transcription start	GCN4_motif: cis-regulatory element involved in endosperm expression	GARE-motif: gibberellin-responsive element	GARE-motif: gibberellin-responsive element
TC-rich repeats: cis-acting element involved in defense and stress responsiveness	HSE: cis-acting element involved in heat stress responsiveness	HSE: cis-acting element involved in heat stress responsiveness	HSE: cis-acting element involved in heat stress responsiveness
TCA-element: cis-acting element involved in SA responsiveness	MBS: MYB binding site involved in drought-inducibility	Skn-1_motif: cis-acting regulatory element required for endosperm expression	MBS: MYB binding site involved in drought-inducibility
TCT-motif: part of a light responsive element	Skn-1_motif: cis-acting regulatory element required for endosperm expression	TATA-box: core promoter element around -30 of transcription start	Skn-1_motif: cis-acting regulatory element required for endosperm expression
Unnamed__4	TATA-box: core promoter element around -30 of transcription start	TCA-element: cis-acting element involved in SA responsiveness	TATA-box: core promoter element around -30 of transcription start
**W box: Binds WRKY type transcription factors**	**W box: Binds WRKY type transcription factors**	**W box: Binds WRKY type transcription factors**	TC-rich repeats: cis-acting element involved in defense and stress responsiveness
chs-Unit 1 m1: part of a light responsive element	TC-rich repeats: cis-acting element involved in defense and stress responsiveness	chs-CMA1a: part of a light responsive element	as-2-box: involved in shoot-specific expression and light responsiveness
Circadian: cis-acting regulatory element involved in circadian control	Circadian: cis-acting regulatory element involved in circadian control	Circadian: cis-acting regulatory element involved in circadian control	Circadian: cis-acting regulatory element involved in circadian control

Table S4. Promoter analysis of *StWRKY1* gene in resistant and susceptible potato genotypes

F06025 WRKY1 promoter	Shepody WRKY1 promoter
ABRE: involved in the abscisic acid responsiveness	ABRE: involved in the abscisic acid responsiveness
ATC-motif: conserved DNA module involved in light responsiveness	ATC-motif: conserved DNA module involved in light responsiveness
Box 4: conserved DNA module involved in light responsiveness	Box 4: conserved DNA module involved in light responsiveness
-	Box I (TTTCAAA): light responsive element
Box II: part of a light responsive element	Box II: part of a light responsive element
-	Box III (CATTTACACT): protein binding site
Box-W1: WRKY1 protein binding site	Box-W1: WRKY1 protein binding site
CAAT-box: cis-acting element in promoter and enhancer regions	CAAT-box: cis-acting element in promoter and enhancer regions
CGTCA-motif: cis-acting regulatory element involved in the MeJA-responsiveness	CGTCA-motif: cis-acting regulatory element involved in the MeJA-responsiveness
G-Box: cis-acting regulatory element involved in light responsiveness	G-Box: cis-acting regulatory element involved in light responsiveness
G-Box: cis-acting regulatory element involved in light responsiveness	G-Box: cis-acting regulatory element involved in light responsiveness
GA-motif: part of a light responsive element	GA-motif: part of a light responsive element
HSE (AGAAAATTCG): cis-acting element involved in heat stress responsiveness	-
I-box: part of a light responsive element	I-box: part of a light responsive element
MBS: MYB Binding Site	MBS: MYB Binding Site
MNF1: light responsive element	MNF1: light responsive element
MRE: MYB binding site involved in light responsiveness<br>O2-site: cis-acting regulatory element involved in zein metabolism regulation	MRE: MYB binding site involved in light responsiveness<br>O2-site: cis-acting regulatory element involved in zein metabolism regulation
Skn-1_motif: cis-acting regulatory element required for endosperm expression	Skn-1_motif: cis-acting regulatory element required for endosperm expression
TATA-box	TATA-box
TATCCAT/C-motif:	TATCCAT/C-motif:
TC-rich repeats: cis-acting element involved in defense and stress responsiveness	TC-rich repeats: cis-acting element involved in defense and stress responsiveness
TGA-element: auxin-responsive element	TGA-element: auxin-responsive element

TGACG-motif: cis-acting regulatory element involved in the MeJA-responsiveness	TGACG-motif: cis-acting regulatory element involved in the MeJA-responsiveness
Unnamed__4	Unnamed__4
Unnamed__5	Unnamed__5
W box: WRKY binding site	W box: WRKY binding site
Circadian: is-acting regulatory element involved in circadian control	Circadian: is-acting regulatory element involved in circadian control

Table S5. The differentially expressed heat shock proteins (HSPs) detected in potato following *P. infestans* inoculation using RNA-seq analysis

Gene name	PGSC ID[a]	Chromosome	FPKM F06025	FPKM Russet Burbank	log2 (fold change)	P value	Q value
Prefoldin chaperone subunit family protein	PGSC0003DMG400001646	1	13.6312	3.48311	1.96847	0.000932	0.032868
Heat shock protein 17.8	PGSC0003DMG400019137	10	17.8939	0.235663	6.2466	$4.88E^{-12}$	$2.7E^{-09}$
HSP26.5 (26.5 kDa heat shock protein, mitochondrial)	PGSC0003DMG400028624	12	2.16571	0.245899	3.1387	0.000958	0.033606

[a] Solanaceae Genomics Resource, Michigan State University, USA.

FPKM, Fragments per kilo base of exon per million fragments mapped; Q value, the false discovery rate (FDR)-adjusted p-value of the test statistic.

F06025- Resistant potato genotype, Russet Burbank- Susceptible potato genotype.

Cufflinks-2.0.1 program was used to find significant changes in transcript expression.
